# Maternal Adiposity in Eastern Libya: Prevalence and Associations with Socioeconomic Characteristics, Unhealthy Eating Behaviours, Physical Inactivity, and Sedentary Behaviour Among Pregnant Women

**DOI:** 10.3390/nu18172828

**Published:** 2026-08-28

**Authors:** Hamdi Lemamsha, Gurch Randhawa

**Affiliations:** 1 Faculty of Health Sciences, University of Tobruk, 2 Alhoria Road, Tobruk T14 H12, Libya; 2 Institute for Health Research, University of Bedfordshire, Putteridge Bury Campus, Hitchin Road, Luton LU2 8LE, UK; gurch.randhawa@beds.ac.uk

**Keywords:** maternal adiposity, pregnancy, prevalence, socioeconomic characteristics, unhealthy eating behaviours, physical inactivity, sedentary behaviour, Libya

## Abstract

**Background/Objectives:** Maternal overweight and obesity are increasing in Libya, yet multicity evidence among pregnant women remains limited, particularly regarding central adiposity and associations with socioeconomic characteristics, unhealthy eating behaviours (UEBs), physical inactivity, and sedentary behaviour. This study estimated the prevalence of women meeting standard WHO BMI cut-offs for overweight/obesity at antenatal assessment and examined associated socioeconomic, dietary, and lifestyle characteristics among women attending participating public antenatal clinics in six Eastern Libyan cities. **Methods:** This cross-sectional study included 3000 pregnant women attending participating public antenatal clinics in six Eastern Libyan cities. A multistage, city-stratified sampling strategy was used. Participants completed a structured Arabic questionnaire assessing socioeconomic characteristics, dietary intake, physical inactivity, and sedentary behaviour. Trained female nurses obtained standardised anthropometric measurements during antenatal assessment using the TANITA RD-545HR Smart Segmental Body Composition Analyser. Body mass index (BMI) was evaluated as an indicator of general pregnancy-period adiposity, while WC and BIA-derived visceral fat rating (VFR) were treated as complementary epidemiological indicators rather than precise or diagnostic measures of visceral adiposity. For each participant, BMI, WC, and VFR were obtained during the same antenatal assessment; however, participants were recruited across different gestational stages. Multivariable linear regression examined associations between the specified exposures and continuous adiposity measures, while modified Poisson regression with robust variance estimated adjusted prevalence ratios (aPRs) for overweight/obesity after adjustment for age, parity, gestational stage, and relevant covariates. **Results:** Among the recruited antenatal clinic sample, 60.6% had BMI-defined overweight/obesity (95% CI: 58.9–62.3); using prespecified descriptive thresholds, 58.9% had WC ≥ 88 cm (95% CI: 57.1–60.7) and 52.1% had BIA-derived VFR ≥ 10 (95% CI: 50.3–53.9). Lower educational attainment (aPR = 1.24, 95% CI: 1.15–1.35, *p* < 0.001) and lower household income (aPR = 1.18, 95% CI: 1.09–1.27, *p* = 0.004) were independently associated with a higher prevalence of overweight/obesity. In the primary scale-level analyses, higher physical inactivity sedentary lifestyle scores (aPR = 1.04, 95% CI: 1.03–1.05, *p* < 0.001) and higher UEBs scores (aPR = 1.07, 95% CI: 1.05–1.09, *p* < 0.001) were associated with a higher prevalence of BMI-defined overweight/obesity. In secondary exploratory item-level analyses, prolonged mobile phone use showed the strongest lifestyle association (aPR = 1.30, 95% CI: 1.20–1.41, *p* < 0.001), while consuming fewer than five daily portions of fruit and vegetables demonstrated the strongest dietary association (aPR = 1.36, 95% CI: 1.24–1.49, *p* = 0.003). The full adjusted models accounted for 6.4–17.6% of variance in the continuous adiposity measures, indicating limited-to-modest explanatory power. **Conclusions:** BMI-defined weight status and elevated pregnancy-period adiposity indicators were common among women attending participating public antenatal services across six Eastern Libyan cities and were associated with socioeconomic disadvantage, sedentary behaviour, and poorer diet quality. The BMI-based classification reflects weight status at antenatal assessment and should not be interpreted as pre-pregnancy overweight/obesity prevalence.

## 1. Introduction

Maternal overweight and obesity remain pressing public health concerns across diverse socioeconomic and geographic contexts, with prevalence increasing steadily over recent decades [1,2,3,4]. A pooled analysis of more than 49 million pregnancies estimated that approximately 43–45% of pregnancies globally involve women with overweight or obesity, while obesity alone affects over 16% of pregnancies [3]. However, most available data come from high-income countries. Eligible population-level evidence from African settings remains limited, reflecting surveillance constraints rather than an absence of burden [3]. Such imbalance restricts contextual interpretation within transitional health systems. Pregnancy involves marked metabolic and endocrine adjustments that may increase vulnerability to excess adiposity and its sequelae [1,5]. Elevated maternal adiposity has been associated with gestational diabetes mellitus, hypertensive disorders of pregnancy, operative delivery, postpartum morbidity, and longer-term cardiometabolic risk affecting both mother and offspring [6,7,8]. These associations indicate that adiposity during pregnancy may operate as both an obstetric and intergenerational exposure.

Body mass index remains the main antenatal indicator for classifying maternal weight status and obesity-related pregnancy risk [9,10,11]. International criteria define underweight as <18.5 kg/m^2^, healthy weight as 18.5–24.9 kg/m^2^, overweight as 25.0–29.9 kg/m^2^, and obesity as ≥30.0 kg/m^2^ [9,10]. Gestational weight gain guidance is also stratified by baseline BMI [10,11]. However, BMI measured during pregnancy differs conceptually from pre-pregnancy BMI because maternal weight increases progressively with gestation through foetal growth, placental development, expansion of maternal blood and extracellular fluid volumes, and changes in maternal body composition. Consequently, absolute BMI measured during pregnancy should be interpreted as maternal anthropometric status at the time of assessment rather than as a direct measure of pre-pregnancy adiposity. Reliance on BMI alone may also inadequately characterise central adiposity or metabolic vulnerability. WC and visceral fat indicators have therefore been considered as complementary anthropometric measures [12,13,14]. Their interpretation during pregnancy also requires caution because uterine enlargement, fluid shifts, and gestational changes in body composition may affect abdominal circumference and bioimpedance-derived estimates, particularly in later pregnancy. Accordingly, WC and visceral fat rating are more appropriately considered supportive epidemiological indicators of pregnancy-period adiposity rather than precise diagnostic measures of central or visceral fat. Evidence nevertheless suggests that central adiposity measures may provide additional information regarding adverse maternal outcomes when interpreted in relation to gestational timing [12,13]. A multidimensional assessment incorporating BMI, WC, and visceral fat indicators may therefore describe complementary aspects of maternal adiposity, provided that gestational stage and the pregnancy-specific limitations of each measure are explicitly considered. Accordingly, throughout the present study, BMI, WC, and BIA-derived VFR are interpreted as pregnancy-period adiposity indicators rather than direct measures of pre-pregnancy obesity or precise diagnostic measures of central or BIA-derived VFR.

Behavioural and socioeconomic characteristics have been associated with maternal adiposity. Physical inactivity and prolonged sedentary behaviour have been associated with general and abdominal obesity [15,16]. Evidence also indicates that sitting time has been associated with adiposity even after accounting for moderate-to-vigorous physical activity [15,16]. Pregnancy-specific evidence has also reported associations involving reduced light-to-moderate movement and extended sedentary exposure [17,18]. Dietary transition in Middle Eastern and North African settings has increased fast-food intake, sugar-sweetened beverages, large portions, and breakfast skipping [19,20]. Lower fruit and vegetable intake further reflects a shift towards obesogenic dietary patterns [19,20]. Evidence from Libyan adult populations demonstrates associations between obesity and several of these behaviours [19,21,22]. However, pregnancy-specific investigations integrating behavioural exposures with multidimensional adiposity indicators remain limited.

The Libyan context presents particular challenges. Rapid urbanisation, prolonged political instability, and lifestyle shifts have coincided with increasing non-communicable disease burden and constrained preventive capacity [23,24,25]. National surveys indicate that more than half of adult Libyan women are affected by overweight or obesity, with female prevalence exceeding that observed among men [19,22]. Such patterns suggest that many women may enter pregnancy with elevated baseline adiposity. Economic analyses estimate that obesity-related conditions accounted for over 1.3 billion Libyan dinars annually in healthcare expenditure prior to recent economic disruption, largely driven by diabetes and cardiovascular disease management [26]. Indirect costs associated with productivity loss and overseas treatment further increase this burden [27]. Fragmented antenatal pathways may restrict early identification and structured lifestyle support.

**Figure 1:**
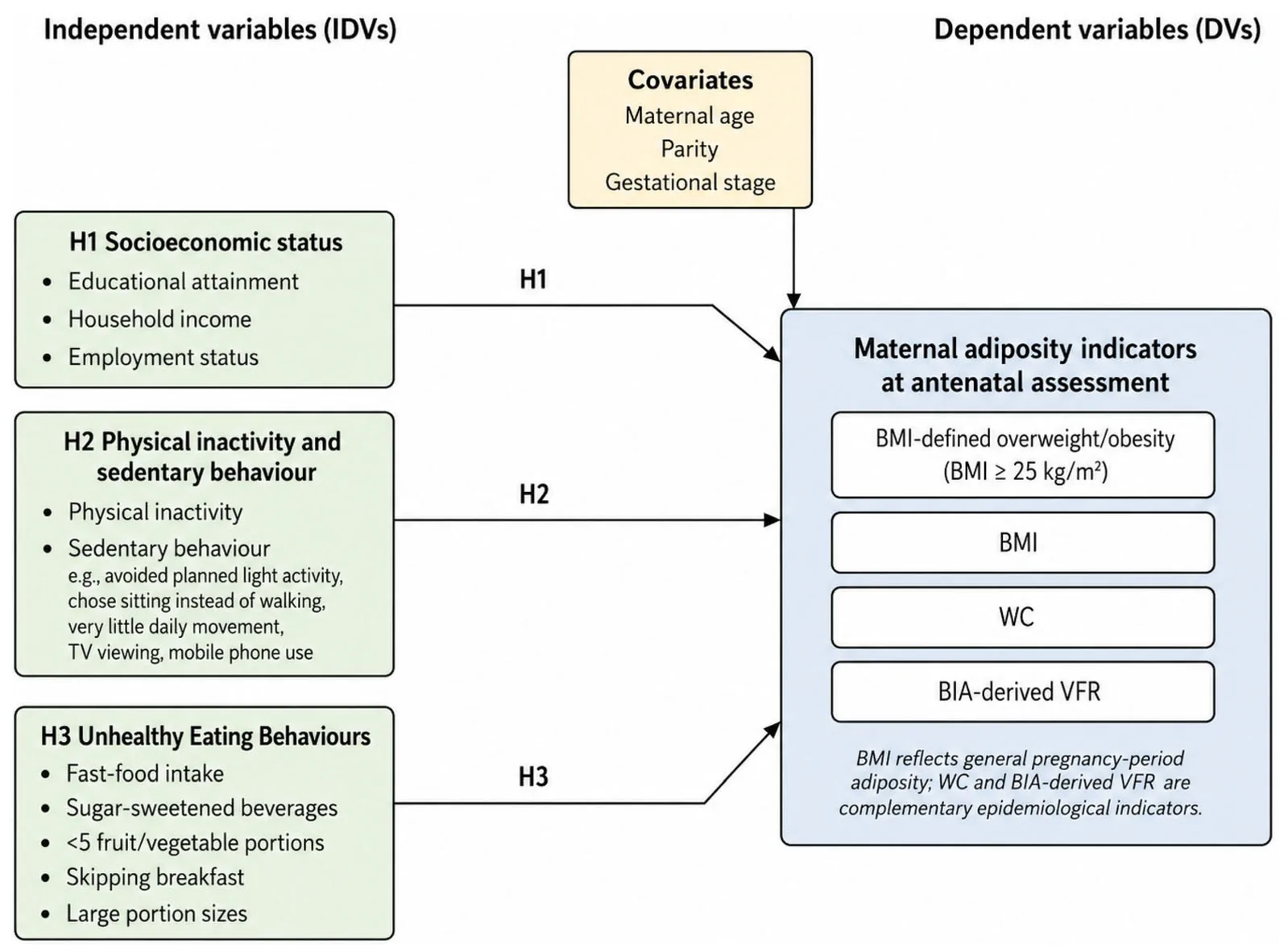
Conceptual framework linking socioeconomic characteristics, dietary behaviours, and lifestyle behaviours with maternal adiposity indicators.

The present study is informed by the Social Ecological Model. The model is used as an organising framework for interpreting maternal adiposity across interconnected contextual and behavioural levels rather than as a formally tested causal model. Education, income, and occupation have been associated with differences in dietary patterns, physical activity, and access to health information, with evidence indicating shifting socioeconomic gradients in obesity among women across transitional settings [28,29,30]. Accordingly, socioeconomic indicators represent broader contextual factors, whereas physical inactivity, sedentary behaviour, and unhealthy eating behaviours (UEBs) represent more proximal behavioural correlates considered within these circumstances [31]. Physical inactivity and sedentary behaviour are behavioural exposures associated with adiposity [15,16], while UEBs include frequent fast-food intake, sugar-sweetened beverages, low fruit and vegetable intake, breakfast skipping, and large portion sizes [20,22,32]. The framework therefore enables the socioeconomic and behavioural associations to be interpreted jointly rather than as isolated correlates, without implying mediation or causal pathways that were not formally tested. Accordingly, H1 examines associations between SES and pregnancy adiposity indicators; H2 examines physical inactivity and sedentary behaviour; and H3 examines UEBs in relation to BMI, WC, and visceral fat indicators (Figure 1).

Recent evidence further indicates that gestational weight gain (GWG) during pregnancy is an important and modifiable determinant of pregnancy outcomes that complements pre-pregnancy BMI. Both inadequate and excessive GWG have been associated with adverse maternal and neonatal outcomes, including hypertensive disorders, caesarean delivery, preterm birth, macrosomia, large-for-gestational-age infants and neonatal intensive care admission. Furthermore, studies suggest that the combination of elevated pre-pregnancy BMI and excessive gestational weight gain confers a substantially greater risk of adverse pregnancy outcomes than either factor alone, highlighting the importance of considering maternal weight both before and during pregnancy [33,34,35,36]. Gestational weight gain was not intentionally excluded from the analysis. Serial maternal weight measurements across pregnancy were not consistently available within the cross-sectional dataset, preventing reliable calculation of individual GWG. GWG is therefore discussed as an important complementary component of maternal weight assessment but was not analysed as an outcome or exposure in the present study.

The main aim of this study is to determine the prevalence of BMI-defined overweight/obesity at antenatal assessment among women attending participating public antenatal clinics in six Eastern Libyan cities. The study also examines associations between maternal adiposity indicators and socioeconomic characteristics, unhealthy eating behaviours, physical inactivity, and sedentary behaviour. Because verified pre-pregnancy BMI and serial gestational weight measurements were unavailable, these anthropometric indicators are interpreted as measures of maternal adiposity during pregnancy rather than as estimates of pre-pregnancy adiposity or gestational weight gain. By integrating multidimensional adiposity markers with behavioural and socioeconomic exposures in a multicity pregnant population in Eastern Libya, the study addresses a gap in pregnancy-specific epidemiological evidence in a context where prior research has largely relied on BMI and general adult samples. Findings from this analysis may contribute to context-specific antenatal risk assessment and may inform more targeted preventive planning and allocation of limited healthcare resources within Libya’s transitional health system.

Figure 1 illustrates the associations of socioeconomic status, physical inactivity and sedentary behaviour, and unhealthy eating behaviours with maternal adiposity. BMI, WC, and BIA-derived VFR represent the adiposity indicators, with age, parity, and gestational stage included as covariates [37,38,39,40,41,42,43,44].

## 2. Materials and Methods

### 2.1. Study Design

A cross-sectional survey design was selected because estimation of BMI-defined overweight and obesity prevalence at antenatal assessment aligns directly with descriptive epidemiological objectives [45]. Cross-sectional approaches permit concurrent assessment of health status, behavioural exposures, and socioeconomic characteristics within defined populations, thereby supporting population-level surveillance and service planning [46]. Because exposures and outcomes were measured simultaneously, findings indicate associations rather than causal relationships [34]. Such designs are operationally feasible for geographically dispersed maternal populations and multi-site investigations [47]. The study was conducted in Eastern and North-Eastern Libya across Benghazi, Derna, Tobruk, Al Bayda, Al Marj, and Ajdabiya, which represent major population and healthcare centres in the region. Regional health analyses indicate variability in urbanisation, socioeconomic conditions, and post-conflict recovery across these cities, factors potentially relevant to maternal health patterns [23,24,25]. Restricting recruitment to a single macro-region supports analytic consistency in a national context where health-system governance may vary [19]. Data collection was undertaken over 18 months, from March 2024 to August 2025, allowing phased site coverage, accommodation of seasonal variation in diet and physical activity, and continuity of recruitment in a setting where service stability may fluctuate.

### 2.2. Study Population

The study population comprised pregnant women residing in Benghazi, Derna, Tobruk, Al Bayda, Al Marj, and Ajdabiya who attended selected public antenatal care facilities during the data-collection period. Recruitment through antenatal clinics ensured alignment with routine maternal health service utilisation and facilitated standardised anthropometric measurement. Eligible participants were women aged 18 years or older with confirmed pregnancy at any gestational stage and residence within one of the six study cities. Participants were required to understand the Arabic-language questionnaire and complete it independently or with assistance from trained female data collectors. Written informed consent was obtained prior to enrolment.

Exclusion criteria were further refined to ensure methodological clarity and minimise potential confounding effects on adiposity measurement. Women with clinically unstable conditions requiring hospital admission or intensive monitoring were excluded because acute illness may alter fluid balance and body composition [3,48,49]. In addition, women with diagnosed endocrine disorders such as uncontrolled thyroid disease or Cushing’s syndrome, as well as chronic systemic conditions including advanced renal or cardiac disease, were excluded due to their established influence on weight regulation and fat distribution [50,51]. Women with physical or cognitive limitations preventing reliable anthropometric measurement or questionnaire completion were also excluded to preserve data quality and internal validity [3,36,45]. These criteria ensured that observed associations reflect typical maternal behavioural and socioeconomic exposures rather than underlying pathological conditions.

### 2.3. Sampling Technique

A multistage, city-stratified sampling strategy was used to obtain broad geographical coverage of pregnant women attending public antenatal services in Eastern Libya, where a complete individual-level sampling frame was unavailable [52,53,54]. Geographically, Libya is commonly divided into three historical regions: Tripolitania in Western Libya, Fezzan in Southern Libya, and Cyrenaica in Eastern Libya [19]. The present study was conducted in Cyrenaica and included Benghazi, Derna, Tobruk, Al Bayda, Al Marj, and Ajdabiya.

In Stage 1 (city stratification), each city was treated as a predefined stratum. An equal target of 500 completed participants per city was intentionally adopted to ensure adequate representation of all six cities and comparable precision for within-city estimation. Proportional allocation was not used because it would have concentrated recruitment in the larger urban centres and yielded much smaller samples from less populous cities. Equal allocation was therefore intended to support balanced multicity comparison rather than population-weighted estimation for Eastern Libya [55].

In Stage 2 (facility selection), three public antenatal facilities were included in each city, giving 18 participating facilities overall. Facilities were selected from operational public antenatal services using predefined feasibility criteria, including adequate antenatal attendance, availability of appropriate space and anthropometric equipment, institutional cooperation, and trained staff able to implement the study protocol consistently. This approach also reflected the local health-system context, in which conflict-related disruption, infrastructure damage, maintenance, and intermittent service availability meant that not all public facilities were consistently able to support the study. Facility selection was therefore eligibility- and feasibility-based rather than probability proportional to size. This distinction is important because probability-based inference requires known selection probabilities, whereas pragmatic facility selection limits generalisation beyond participating services [54]. Facility selection was therefore not probability proportional to size (PPS), and no population sampling weights were applied. Consequently, pooled estimates describe the recruited multicity public antenatal clinic sample and should not be interpreted as population-weighted prevalence estimates for all pregnant women in Eastern Libya [52,54].

In Stage 3, eligible pregnant women attending participating facilities were recruited using systematic random sampling from routine attendance lists, with a random starting point. The sampling interval was calculated as $k=N_{s}/n_{s}$, where $N_{s}$ represented the number of eligible women available during the relevant recruitment period and $n_{s}$ represented the number required. Every kth eligible woman was invited until the allocated target was reached [56].

#### Recruitment and Response Rate

The final sample comprised 3000 pregnant women, with 500 participants recruited from each of the six cities across 18 public antenatal facilities. Equal city allocation was used to provide balanced geographical representation and comparable within-city precision rather than population-proportional estimation. Recruitment and questionnaire completion were supervised by trained nurses, who checked questionnaires for completeness during data collection.

**Figure 2:**
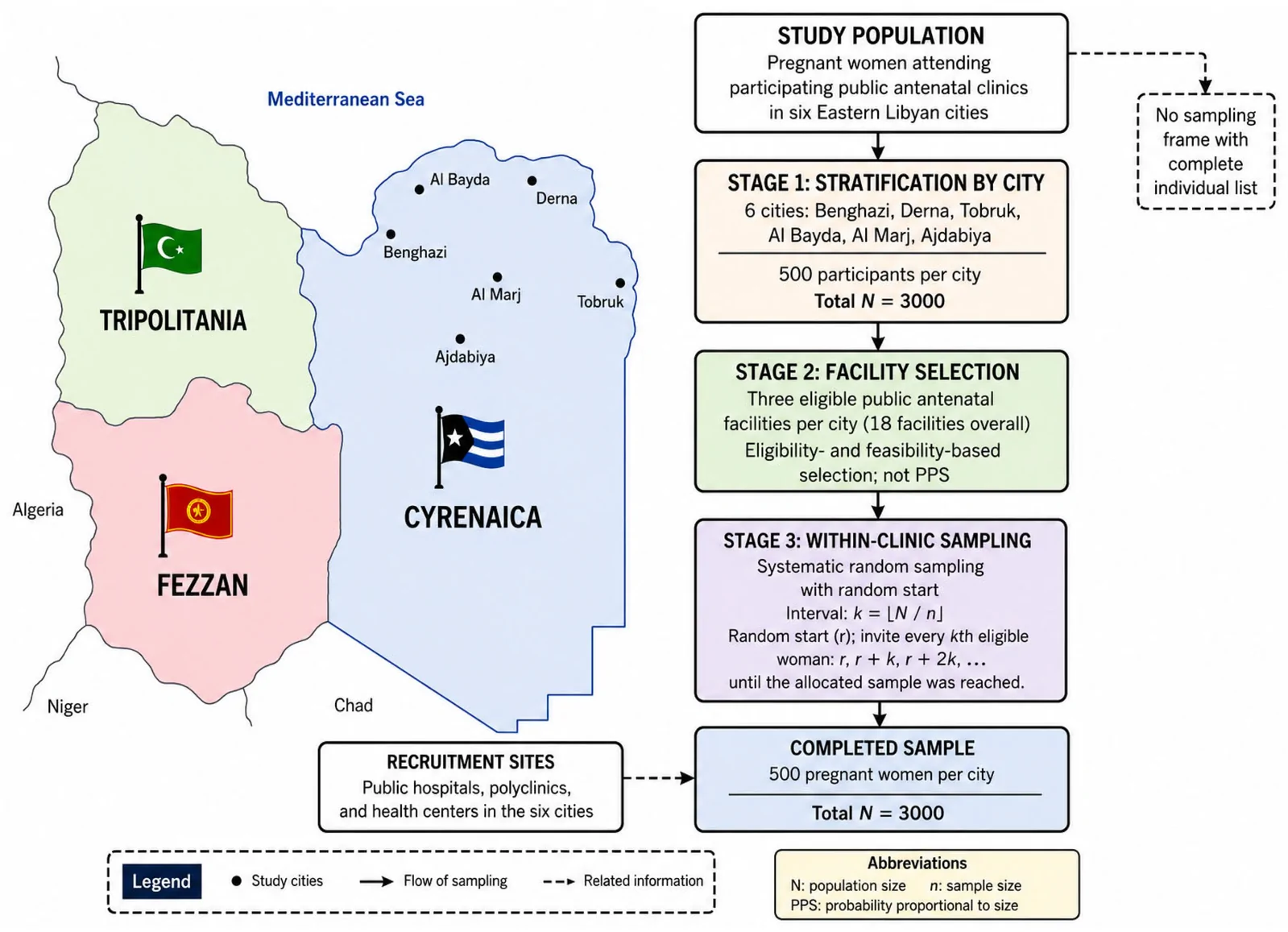
Multistage city-stratified sampling of pregnant women attending public antenatal facilities in Eastern Libya.

A total of 3192 eligible women were approached, of whom 3000 completed participation, giving an overall response rate of 94.0%. As shown in Table 1, response rates varied modestly across cities: 91.6% in Tobruk, 92.6% in Derna, 94.5% in Al Marj, 94.7% in Al Bayda, 95.2% in Benghazi, and 95.4% in Ajdabiya. The consistently high participation across cities reduced, although did not eliminate, the potential for non-response bias. The multistage recruitment process is summarised in Figure 2.

$$\mathrm{Overall}\;\mathrm{response}\;\mathrm{rate}=\frac{3000}{3192}\times 100=93.98\%\approx 94.0\%$$

Six city strata, with three participating public antenatal facilities per city (18 facilities). Systematic random recruitment within facilities yielded 500 participants per city and a final sample of 3000 women.

### 2.4. Sample Size

Sample size was determined using the single-proportion formula. A 95% confidence level, expected prevalence of 50%, and 5% margin of error were applied. This yielded a minimum of 384 participants per city [48,49]. The target was increased to 500 per city, giving a total sample of 3000. The calculation was applied at the city-stratum level, and the increase from 384 to 500 participants per city was intended to improve precision and provide sufficient observations for multivariable modelling rather than to represent a formal cluster design-effect adjustment. Equal allocation provided comparable precision across the six cities and adequate observations for multivariable analysis. It was not intended to reflect differences in city population size.

Potential clustering of participants within antenatal facilities was recognised because women attending the same facility may share characteristics that introduce intra-facility correlation [57]. No reliable outcome-specific intra-cluster correlation coefficient (ICC) was available for comparable Libyan antenatal facilities at the design stage. Consequently, an ICC-based design effect (DEFF) was not incorporated into the a priori sample-size calculation. The increase to 500 participants per city was therefore a pragmatic inflation for improved precision and model stability rather than a formal DEFF adjustment [58,59]. Potential intra-facility correlation was subsequently accounted for analytically using clinic-level clustering and design-adjusted variance estimation, as described in Section 2.7. Equal city allocation supported balanced multicity coverage rather than population-proportional estimation. Consequently, pooled estimates represent the recruited multicity antenatal sample and should not be interpreted as population-weighted prevalence estimates for all pregnant women in Eastern Libya.

### 2.5. Measures and Instrumentation

#### 2.5.1. Questionnaire Design

Scale 1—Demographic information

The demographic scale was adapted from prior maternal and obesity research conducted in Libya [19,26,60] and structured in accordance with the WHO STEPS framework for sociodemographic profiling in non-communicable disease research [61]. Variables included age group, marital status, parity, residence type, and pregnancy characteristics. Categorisation reflected distributions previously reported among Libyan women of reproductive age [50], thereby supporting contextual comparability while maintaining relevance to pregnancy research in Eastern Libya.

Scale 2—Socioeconomic status (SES)

The SES scale was derived from education–income–occupation frameworks applied in earlier Libyan obesity studies [19,22,26,50] and aligned with WHO STEPS indicators [57]. Educational attainment, employment status, occupation category, and household income were retained as core measures. Perceived financial strain and housing tenure were incorporated to capture contextual economic vulnerability during pregnancy. This configuration enables examination of socioeconomic gradients in maternal overweight and obesity while preserving analytical continuity with national evidence. Scale 1 and Scale 2 were analysed as categorical and formative variables rather than summed reflective scales. Demographic items were used to describe the study population and support adjustment for age, gestational stage, parity, residence, and pregnancy type. Socioeconomic indicators were analysed individually because education, income, employment, occupation, housing tenure, household size, and perceived financial strain represent related but non-interchangeable dimensions of socioeconomic position.

Scale 3—Physical Inactivity and Sedentary Lifestyle Scale

The Physical Inactivity and Sedentary Lifestyle Scale measured reduced movement and prolonged sitting during pregnancy. Item development was informed by evidence linking inactivity and sedentary behaviour with overweight and abdominal adiposity [15,16,62]. Adaptation for pregnant populations followed established methodological considerations for physical activity assessment during gestation [63]. The instrument comprised 12 items organised into two domains—physical inactivity and sedentary behaviour—using a five-point frequency response format to quantify habitual exposure. Scale 3 was scored by assigning numerical values from 1 to 5, where 1 represented “Never” and 5 represented “Very often”. Item scores were summed to generate a total physical inactivity and sedentary lifestyle score ranging from 12 to 60, with higher scores indicating greater self-reported inactivity and sedentary exposure. The total Physical Inactivity and Sedentary Lifestyle composite score was specified as the primary exposure for H2 because it represented the psychometrically evaluated construct. Domain- and item-level analyses were retained as secondary exploratory analyses to identify specific behaviours underlying the overall scale-level association. Domain scores were also calculated separately, with the six physical inactivity items ranging from 6 to 30 and the six sedentary behaviour items ranging from 6 to 30. Summed or averaged composite scores are commonly used when several Likert-type items assess a shared construct, although such scores should be interpreted as structured questionnaire indicators rather than direct behavioural measurements [64,65]. Because the total and domain scores aggregated multiple ordered items and demonstrated acceptable internal consistency, they were treated as approximately continuous for parametric analysis. Individual five-point items were coded from 1 to 5 as ordered frequency scores to estimate the linear trend associated with increasing behavioural frequency, rather than being interpreted as true interval-level measurements. For individual physical inactivity and sedentary-behaviour items, the reported β coefficients and aPRs therefore represent the association corresponding to a one-category increase on the five-point response scale, rather than comparisons between predefined categories.

Scale 4—Unhealthy Eating Behaviours (UEBs) Scale

The UEBs scale assessed dietary behaviours associated with excess energy intake. Item selection was informed by Libyan research linking fast-food intake, sugar-sweetened beverages, and meal skipping with obesity [22], alongside primary care screening tools for obesogenic eating patterns [66]. The five items were treated as behavioural screening indicators rather than quantitative estimates of habitual dietary intake. They assessed consumption of fewer than five fruit and vegetable portions, large portion sizes, sugar-sweetened beverage consumption, fast-food intake, and breakfast skipping. Responses were recorded using a five-point frequency scale, scored from 1 (“Never”) to 5 (“Daily”). The total UEBs score ranged from 5 to 25, with higher scores indicating more frequent UEBs. The total UEBs composite score was specified as the primary exposure for H3 because it represented the psychometrically evaluated overall behavioural construct. Individual dietary items were examined secondarily to identify which specific behaviours contributed most strongly to the scale-level association. The composite score therefore reflected the frequency of these predefined behaviours and was not interpreted as an estimate of energy or nutrient intake. Equal weighting was applied because all items represented core obesogenic behaviours identified in the study framework. No reverse scoring was required because all items were coded in the same direction [56,67]. For inferential analysis, the summed multi-item score was treated as approximately continuous, while individual five-point responses were analysed as ordered 1–5 frequency scores representing increasing behavioural exposure. This coding was used to estimate trend associations and did not imply that the response categories constituted precise quantitative measures of dietary intake. Accordingly, effect estimates for individual UEB items represent a one-category increase on the 1–5 frequency scale rather than comparisons between predefined response groups.

The questionnaire was administered in Arabic and adapted from established obesity, dietary, physical activity, and maternal health instruments. Content validity was reviewed by specialists in public health, nutrition, maternal health, and epidemiology. A pilot assessment among pregnant women evaluated clarity, comprehension, flow, and administration procedures, leading to minor wording refinements. Trained female nurses administered the questionnaire using standardised procedures and checked completed forms immediately to minimise missing data. Behavioural items used defined recall periods to reduce recall bias: the previous 7 days for physical inactivity and sedentary behaviour and the previous month for unhealthy eating behaviours [68,69]. Closed response categories were used to improve response consistency and scoring.

#### 2.5.2. Anthropometric Measurements and Instrumentation

Anthropometric measurements were obtained by trained nurses using standardised procedures across participating facilities. Height was measured barefoot to the nearest 0.1 cm using a portable stadiometer. Body weight, body fat percentage, visceral fat rating (VFR), and segmental body composition were assessed using the TANITA RD-545HR Smart Segmental Body Composition Analyser (TANITA Corporation, Tokyo, Japan) (bioelectrical impedance), with weight recorded to the nearest 0.1 kg. Each participating facility was equipped with the same model of TANITA RD-545HR analyser and measurements were performed by trained operators using a common written protocol. Equipment was checked before field deployment and subsequently verified weekly throughout data collection. Device zeroing, weighing performance, electrode/contact surfaces, and general operating condition were checked. Any unit showing an unacceptable discrepancy was withheld from measurement until the problem was resolved. This cross-site quality-control procedure was used to minimise systematic measurement variation between the 18 participating facilities.

The use of the same analyser model, common operating procedures, trained personnel, and repeated equipment verification was particularly important in this multicentre study. Modern BIA provides a rapid, non-invasive and practical approach to body-composition assessment, while segmental configurations permit regional assessment beyond conventional whole-body measurements [70,71]. Nevertheless, device characteristics and measurement conditions can affect BIA estimates. Standardisation across sites was therefore prioritised rather than assuming interchangeability of measurements obtained under different conditions [72]. Segmental BIA has advantages over whole-body approaches because individual body regions can be assessed separately, while BIA is non-invasive and repeatable. Modern multifrequency and segmental configurations have also improved regional body-composition assessment. Early pregnancy assessment (≤12–14 weeks) was prioritised where feasible to reduce gestational distortion of central adiposity indicators. This timing was methodologically important because WC is considered more interpretable in early pregnancy, particularly before approximately 16–20 weeks’ gestation, when uterine enlargement has less influence on abdominal measurement before the uterus reaches the umbilical level [73].

Maternal height was measured once using a calibrated stadiometer, and body weight was measured using the TANITA RD-545HR at enrolment. Height was entered into the analyser, which automatically generated BMI, body fat percentage, VFR, and other body composition measurements. BMI (kg/m^2^) was calculated from measured weight and height. VFR was generated as a device-derived rating. Adult manufacturer ranges classify 1–12.5 as healthy and 13–59 as excessive [41,70,71]; because these ranges are not validated in pregnancy, VFR was analysed continuously. BMI reflected anthropometric status at antenatal assessment rather than pre-pregnancy BMI; therefore, WHO BMI categories were applied descriptively. Gestational age was included as a covariate to reduce, but not eliminate, variation related to gestational weight gain, uterine enlargement, fluid redistribution, and body-composition changes. Pre-pregnancy BMI remains widely used in pregnancy research [74,75]; however, verified pre-pregnancy weight was unavailable for all participants. BMI at enrolment was therefore interpreted as a pregnancy-period adiposity indicator rather than pre-pregnancy BMI. Serial pregnancy weights were also unavailable; therefore, gestational weight gain could not be calculated. Consequently, enrolment BMI may reflect both pre-existing adiposity and physiological gestational weight gain.

Bioelectrical impedance measurements followed the same pre-measurement protocol across facilities. Participants were measured barefoot, in light clothing, using the same standing position and electrode-contact procedure. Measurements were undertaken before anthropometric data were recorded in the study questionnaire. Participants were instructed to avoid conditions likely to produce substantial short-term alteration in body water before assessment, and measurements were deferred when the operator considered the participant unsuitable for reliable measurement. These procedures were applied consistently by the trained nurses and operators across sites. This standardisation is important because BIA depends strongly on body-water distribution, and hydration is a recognised constraint of the method [71]. Recent exercise, dehydration and food consumption can also alter BIA measurements.

Gestational weight gain ranges were interpreted using Institute of Medicine guidance [9]. WC and visceral fat were analysed as indicators of central adiposity, acknowledging pregnancy-specific interpretive considerations [9,10,11,13]. These gestational weight gain ranges were retained for contextual reference only; individual gestational weight gain was not calculated or analysed because serial pregnancy weights were unavailable. Neither WC nor impedance-derived VFR was interpreted as a standalone diagnostic measure during pregnancy. Instead, they were used alongside BMI as complementary epidemiological indicators of maternal adiposity. This approach reflects evidence that BMI, WC, and BIA-derived VFR capture related but distinct dimensions of adiposity associated with maternal and neonatal outcomes [37,76,77]. WC ≥ 88 cm was retained as an adult-based descriptive epidemiological threshold because this level is associated with substantially increased metabolic risk in adult women; however, it was not interpreted as a validated pregnancy-specific diagnostic cut-off [13,38,39].

Integration of BMI and WC reflects evidence that these measures capture complementary aspects of maternal adiposity [13,38]. Combined indicators may reduce the limitations associated with reliance on a single anthropometric measure [12,39]. A meta-analysis of 29 studies involving 56,438 pregnant women found greater WC and visceral adipose tissue depth among women with gestational diabetes mellitus [68]. Second-trimester evidence has also demonstrated associations between maternal BIA-derived VFR and foetal biometric measures after adjustment for gestational age [69].

Application of BIA enabled assessment of body-composition characteristics not captured by BMI alone [40,41]. Contemporary BIA is widely used in clinical and research settings because it is non-invasive, rapid, accessible and radiation-free [77]. Evidence also supports the value of segmental impedance approaches for regional body-composition assessment [76]. However, BIA-derived estimates remain indirect and device-dependent. Oliver et al. [72] reported substantial individual-level disagreement between BIA and four-compartment reference estimates in non-pregnant adults. Their findings reinforce the need for standardised measurement and cautious interpretation rather than supporting interchangeability with criterion methods.

Previous pregnancy studies have used BIA during antenatal assessment and reported associations between BIA-derived VFR, metabolic indicators and pregnancy outcomes [42,43]. Nevertheless, WC and impedance-derived VFR were interpreted cautiously, particularly during later gestation. Physiological fluid shifts, uterine enlargement and altered fat distribution may influence abdominal and impedance measurements [42,43]. Anatomical measurement site and gestational timing are also important considerations [44]. Accordingly, BIA-derived VFR and WC were treated as complementary epidemiological indicators rather than criterion measurements of visceral adipose tissue. Interpretation focused on relative adiposity differences rather than clinical diagnosis.

Women with underweight were retained in descriptive analyses, whereas inferential models focused on overweight and obesity (BMI ≥ 25.0 kg/m^2^). Anthropometric classification thresholds and recommended gestational weight gain ranges are summarised in Table 2.

Application of bioelectrical impedance analysis was selected to enable direct assessment of body composition, including fat mass and BIA-derived VFR, which are not captured by conventional anthropometric indices. Evidence supports its reliability and acceptable validity in clinical and population settings, with strong agreement reported against reference methods and high reproducibility across repeated measurements [62]. In pregnancy, bioelectrical impedance offers a non-invasive and radiation-free approach suitable for repeated assessment and has been applied in longitudinal maternal studies to monitor physiological changes in body composition [43]. Standardised pre-measurement conditions, including control of hydration status and consistent measurement protocols, were implemented to minimise variability and enhance measurement accuracy [44]. The use of bioelectrical impedance analysis was therefore retained because previous pregnancy studies have used multifrequency bioelectrical impedance devices during antenatal care and reported associations between visceral fat mass, fasting glucose, HbA1c, gestational diabetes mellitus, leptin concentration, and infant birth weight [79,80]. Evidence from middle and late pregnancy cohorts has also indicated that bioelectrical impedance-derived body fat percentage may predict adverse outcomes, including abnormal blood pressure, anaemia, and operative delivery among women with gestational diabetes [81].

Nevertheless, interpretation of WC and impedance-derived visceral fat was undertaken cautiously, especially among women measured during the second and third trimesters. Physiological fluid shifts, pregnancy-related changes in hydration status, uterine enlargement, and altered fat distribution may influence impedance-derived estimates and abdominal measurements during later gestation [43,66]. Although BIA-derived VFR assessment has been successfully applied across gestation using ultrasound methods, anatomical measurement site and gestational timing remain important determinants of validity and comparability [82]. Accordingly, WC and BIA-derived VFR were interpreted as complementary epidemiological indicators of maternal adiposity during pregnancy rather than precise diagnostic measures of abdominal or BIA-derived VFR. For epidemiological comparability, WC ≥ 88 cm was retained as the established adult female threshold associated with substantially increased metabolic risk, but was not considered a validated pregnancy-specific diagnostic cut-off. Interpretation focused on relative adiposity differences rather than clinical diagnosis [37,43,44,79,80]. Women with underweight were retained in descriptive analyses, whereas inferential models focused on overweight and obesity (BMI ≥ 25.0 kg/m^2^).

#### 2.5.3. Measurement Validity and Reliability

Psychometric evaluation was conducted prior to hypothesis testing to confirm measurement adequacy. Face validity demonstrated substantial inter-rater agreement (Cohen’s κ = 0.74–0.82), exceeding recommended thresholds [49]. Content validity ratios ranged from 0.78 to 0.92, surpassing the critical value for a 10-member expert panel [83]. Construct validity of behavioural scales was examined using exploratory factor analysis. The Physical Inactivity and Sedentary Lifestyle Scale demonstrated strong sampling adequacy (KMO = 0.87), with significant Bartlett’s test (χ^2^(66) = 1485.32, *p* < 0.001), yielding a two-factor structure explaining 59.20% of variance. The UEBs Scale showed adequate factorability (KMO = 0.81; χ^2^(10) = 612.45, *p* < 0.001), explaining 57.10% of variance. Internal consistency reliability was satisfactory (Cronbach’s α = 0.86 and 0.79), exceeding the ≥0.70 criterion [23,84]. Temporal stability of the two behavioural scales was additionally evaluated by repeat administration in the pilot subsample. Test–retest reliability was assessed using the intraclass correlation coefficient (ICC) with 95% confidence intervals, an appropriate approach for evaluating agreement between repeated measurements [85,86]. The Physical Inactivity and Sedentary Lifestyle Scale demonstrated good temporal reliability (ICC = 0.88, 95% CI: 0.82–0.92), as did the UEBs Scale (ICC = 0.83, 95% CI: 0.75–0.89). Reliability estimates should be interpreted with consideration of the retest sample size and associated precision [87]. Demographic and socioeconomic measures were treated as formative indicators for which internal consistency testing was not conceptually required. Psychometric characteristics are summarised in Table 3.

Hunot-Alexander et al. [85] similarly distinguished internal consistency from temporal stability and assessed the latter through ICC following repeated questionnaire administration.

### 2.6. Recruitment Procedure

Following ethical approval, formal authorisation was obtained from the Director of Health Administration Services in each participating city. Official correspondence was submitted to municipal health authorities in Benghazi, Derna, Ajdabiya, Al Marj, Al Bayda, and Tobruk. After administrative approval, data collection was authorised within selected public hospitals, polyclinic healthcare centres, and health centres.

Recruitment sites included Benghazi Medical Centre, Al-Sabri Polyclinic Healthcare, and Al-Keish Polyclinic Healthcare in Benghazi; Alwahda Hospital, Yousef Borhail Polyclinic Healthcare, and Almarhoum Mahmoud Lahreesh Polyclinic Healthcare in Derna; Alshaheed Mohammed Al-Megreif Hospital, Ajdabiya Polyclinic Healthcare Complex, and Sultan Health Centre in Ajdabiya; Al Marj Hospital, Al Marj Eastern Health Centre, and Al Marj Western Health Centre in Al Marj; Al Bayda Medical Centre, Polyclinic Healthcare 1, and Polyclinic Healthcare 4 in Al Bayda; and Tobruk Medical Centre, Mokhtar Polyclinic Healthcare, and Al-Jihad Clinic Healthcare in Tobruk. Facility directors provided space and logistical support for questionnaire administration and anthropometric measurement.

Three public antenatal facilities were included in each city, giving 18 facilities overall. Site selection considered antenatal service availability, geographical coverage, operational capacity, availability of the required anthropometric equipment and trained personnel, and institutional cooperation with the field protocol. This was particularly relevant because the availability and operational status of public health facilities varied across the study area. The same eligibility criteria, recruitment procedure, questionnaire, anthropometric protocol, and TANITA analyser model were used across all participating facilities to maximise procedural comparability.

Forward–back translation was completed before fieldwork to support linguistic accuracy and conceptual equivalence [88,89]. Two independent certified Libyan translators translated the questionnaire and study documents from English into Arabic. A harmonised Arabic version was produced by comparison and consensus. Two independent bilingual translators, blinded to the original version, then back-translated the documents into English. An expert committee comprising a maternal health specialist, public health researcher, Arabic-language proofreader, and the translators reviewed semantic and conceptual equivalence. Minor discrepancies were resolved before psychometric testing and field deployment.

Trained female nurses were allocated to each site under field supervision. They received structured training in eligibility assessment, informed consent, questionnaire administration, confidentiality, recruitment-log completion, and anthropometric procedures. Questionnaire completion was supervised, and returned forms were checked immediately for completeness. Unintentionally omitted items were clarified, where ethically appropriate, before participants left the facility. Responses that could not be clarified were coded as missing rather than imputed.

Systematic recruitment was conducted among eligible pregnant women attending antenatal services until 500 completed participants were obtained in each city. Participant identification was guided by antenatal attendance registers and clinic logbooks rather than open convenience recruitment. These records covered women receiving routine antenatal follow-up from different residential areas and facility catchment zones. Eligible women were approached according to clinic attendance flow using the same predefined procedure. This reduced interviewer discretion and supported consistent application of the eligibility criteria. Recruitment across hospitals, polyclinic healthcare centres, and health centres also reduced dependence on a single facility [90,91].

Public antenatal services constituted the study sampling frame. Women attending private antenatal services exclusively were outside the sampling frame. No comparative data were collected to determine whether they differed from recruited women in socioeconomic characteristics, healthcare utilisation, or adiposity. We therefore make no assumption that public- and private-service populations were equivalent.

Recruitment through multiple public facilities was intended to broaden coverage of the public antenatal population and reduce single-facility selection effects. Nevertheless, healthcare-based sampling may under-represent women who use private care exclusively, initiate antenatal care late, attend irregularly, or experience barriers to public healthcare access [42,66,92,93]. This residual selection bias cannot be eliminated by multicentre recruitment. Accordingly, the findings are interpreted primarily as representing pregnant women attending participating public antenatal services in the six Eastern Libyan cities during the study period, rather than all pregnant women in Eastern Libya.

### 2.7. Data Analysis Technique

Quantitative analyses were conducted using IBM SPSS Statistics version 29.0 (IBM Corp., Armonk, NY, USA). IBM SPSS Amos version 29.0 was used only to prepare the conceptual framework shown in Figure 1; no structural equation modelling (SEM) or confirmatory factor analysis (CFA) was performed. Completed questionnaires were checked against field logs before analysis. Missing responses were coded as missing rather than imputed, and complete-case analysis was applied for models requiring all relevant variables [40,41]. Missingness was assessed for each outcome, exposure, and covariate and across gestational trimesters. No missing data were present for the major outcomes, covariates, composite scores, or secondary behavioural and dietary variables (0/3000; 0.0%). Accordingly, all H1–H3 regression models used the full complete-case sample (*n* = 3000). The primary H1–H3 models used complete-case samples of *n* = 3000; missing values were not imputed. Complete-case analysis was interpreted cautiously because systematic missingness may introduce bias [94,95]. Descriptive statistics summarised demographic, socioeconomic, lifestyle, dietary, and anthropometric variables. Continuous variables were reported as means and standard deviations, while categorical variables were presented as frequencies and percentages. Normality was assessed using Shapiro–Wilk tests, Q–Q plots, and skewness and kurtosis values [96]. Given the large sample size (*n* = 3000) and approximately symmetrical distributions, parametric procedures were considered appropriate. For Likert-derived variables, summed multi-item scores were treated as approximately continuous because they aggregated multiple ordered responses and showed satisfactory psychometric properties. Individual five-point items were entered as ordered scores coded from 1 to 5 to estimate a linear trend across increasing response frequency. For these items, each regression coefficient or aPR represents the association corresponding to a one-category increase in the five-point response scale. The 1–5 coding was therefore used as an ordinal-trend parameterisation rather than to imply that the response categories were intrinsically interval. The appropriateness of the parametric models was further evaluated through the stated linearity and residual diagnostics.

Hypothesis testing was conducted using Pearson correlation, adjusted multivariable linear regression, and modified Poisson regression with robust variance. Modified Poisson regression was selected because it provides direct estimates of prevalence ratios, which are generally recommended for cross-sectional studies with binary outcomes, particularly when the outcome may not be rare. In contrast, odds ratios derived from logistic regression may overestimate the magnitude of association when outcomes are common, potentially reducing the interpretability of the findings. Maternal age, parity, and gestational stage were specified a priori as covariates because of their relationships with maternal anthropometric status. Because the binary overweight/obesity outcome was modelled using modified Poisson regression rather than logistic regression, the Hosmer–Lemeshow test was not applied. Covariates were specified a priori, based on established confounding relationships and the conceptual framework (Figure 1), rather than statistical significance. Age and parity were included because of their relationships with maternal adiposity and socioeconomic/behavioural exposures; gestational stage because pregnancy progression affects weight, WC, and body composition; and SES in H2–H3 because it may relate to both behavioural exposures and adiposity. Gestational stage was modelled categorically (first, second, and third trimester), with the first trimester as reference. Gestational age was not modelled continuously; therefore, no linear relationship across gestational weeks was assumed. Adjustment for gestational stage reduced, but could not eliminate, the influence of pregnancy progression on BMI, WC, and BIA-derived VFR. Verified pre-pregnancy BMI and serial weights required to calculate gestational weight gain were unavailable. Smoking status, previous gestational diabetes, hypertensive disorders, and other pregnancy complications were also not consistently recorded. Residual confounding by these factors cannot therefore be excluded, and adjusted estimates were interpreted as associations rather than causal effects. Accordingly, the models could not adjust directly for pre-pregnancy BMI or gestational weight gain. Adjustment for gestational stage was used to reduce pregnancy-stage-related variation but should not be interpreted as equivalent to controlling for these unavailable maternal weight characteristics.

For H1, socioeconomic indicators were examined as independent variables, including educational level, household income, and employment status. BMI, WC, and VFR were entered separately as continuous dependent variables in adjusted linear regression models, controlling for age, parity, and gestational stage. Modified Poisson regression with robust variance was then used to examine overweight/obesity status, defined as BMI ≥ 25 kg/m^2^, with adjusted prevalence ratios (aPRs), 95% confidence intervals, and *p*-values reported. For H2, the total Physical Inactivity and Sedentary Lifestyle composite score was analysed as the primary exposure using Pearson correlation, adjusted linear regression for BMI, WC, and VFR, and modified Poisson regression with robust variance for BMI-defined overweight/obesity. Models were adjusted for age, parity, gestational stage, and SES. Individual physical inactivity and sedentary behaviour items were subsequently examined as secondary exploratory exposures to identify specific behavioural patterns contributing to the overall association. For H3, the total UEBs composite score was similarly analysed as the primary exposure, followed by secondary item-level analyses of fast-food intake, sugar-sweetened beverages, low fruit and vegetable intake, breakfast skipping, and large portion sizes. H3 models were adjusted for age, parity, gestational stage, and SES. Statistical significance was set at *p* < 0.05. Multiplicity was additionally assessed using the Benjamini–Hochberg false discovery rate (FDR) procedure across the 22 H1–H3 predictor tests for BMI-defined overweight/obesity, with q < 0.05 considered significant. Composite scores remained the primary H2 and H3 exposures, while item-level analyses were treated as exploratory. No omnibus multiplicity adjustment was applied across the correlated BMI, WC, and VFR outcomes; these associations were therefore interpreted cautiously. Gestational trimester was tested as an effect modifier by including interaction terms between trimester and each exposure in the adjusted BMI, WC, VFR, and overweight/obesity models.

Gestational trimester was additionally examined as a potential effect modifier using trimester × predictor interaction terms in the adjusted BMI, WC, VFR, and BMI-defined overweight/obesity models. Interaction *p*-values and trimester-specific estimates with 95% CIs were examined where appropriate [97,98]. These analyses were undertaken to assess whether the magnitude of the observed socioeconomic, behavioural, and dietary associations differed across gestational stages rather than assuming that the associations were constant throughout pregnancy. Regression modelling matched the study outcomes and controlled for confounding. Linear regression was used for continuous adiposity indicators (BMI, WC, and VFR), whereas modified Poisson regression with robust variance was used for the binary overweight/obesity outcome to estimate adjusted prevalence ratios. Pearson correlation examined initial bivariate associations, and adjusted R^2^ values were used to summarise the overall explanatory power of each full adjusted linear regression model, including both exposure variables and covariates; they were not interpreted as variance attributable to any single predictor or predictor group. Multivariable models adjusted for predefined covariates, including age, parity, and gestational stage, although residual confounding may remain in observational research [99,100]. Robust variance estimation was applied to obtain valid standard errors and confidence intervals for prevalence ratio estimates, consistent with current epidemiological recommendations for cross-sectional analyses of binary outcomes. Model diagnostics included VIF and tolerance statistics for multicollinearity, residual plots for linearity and homoscedasticity, assessment of model specification, and Cook’s distance for influential observations. Sensitivity analyses across BMI, WC, and VFR assessed the consistency of associations. Sensitivity analyses repeated the H1–H3 models among first-trimester participants (*n* = 660), omitting gestational stage while retaining the remaining covariates. Robustness was assessed by comparing the direction, magnitude, and 95% CIs with the full-sample estimates [97,98]. Restriction to the first trimester provided an additional assessment of robustness in the subgroup least affected by later gestational increases in maternal weight, uterine size, and pregnancy-related changes in body composition.

The multistage sampling design was incorporated into the analytical plan. Clinic was specified as the primary sampling unit, and city was treated as the stratification variable [55,56]. The design comprised six city strata and 18 clinic clusters; no population sampling weights were applied. Continuous BMI, WC, and VFR models used SPSS Complex Samples General Linear Models to obtain design-adjusted standard errors and 95% CIs [9,57]. Modified Poisson models for BMI-defined overweight/obesity used GENLIN generalised estimating equations with clinic-level clustering, robust covariance estimation, and city included as a fixed factor. Regression coefficients and adjusted prevalence ratios were interpreted as measures of association rather than causality because of the cross-sectional design [45,47]

## 3. Findings

### 3.1. Participant Characteristics

Table 4 presents the demographic profile of pregnant women recruited across six cities in Eastern Libya (*n* = 3000), with equal allocation of 500 participants per city. Women aged 25–29 years represented the largest age group (24%), closely followed by those aged 35–39 years (24%), whereas women aged ≥40 years comprised only 7%, indicating concentration within peak reproductive age categories. The second trimester accounted for the highest proportion of participants (41%), suggesting active engagement with antenatal services during mid-pregnancy. Married women constituted 96% of the sample, reflecting demographic stability within the cohort. Parity of 2–3 previous births was most frequent (34%), while nulliparous women represented 22%. Urban residence predominated (64%), with rural residence accounting for 13%. Singleton pregnancies were overwhelmingly reported (97%). The concentration of participants within married, urban, and multiparous categories provides contextual grounding for subsequent obesity analyses.

### 3.2. Socioeconomic Characteristics

Table 5 summarises the socioeconomic characteristics of participants. University education was the most common level (60.3%), followed by secondary education (20.0%). Employment was reported by 42.0%, while 31.0% were homemakers and 9.0% were unemployed. Monthly income was most commonly 2000–2999 LYD (33.0%), whereas 9.0% reported less than 1000 LYD. Most participants lived in households of 3–4 members (52.0%). Regarding financial strain, 60.0% were comfortable, 30.0% coping, 8.0% difficult, and 2.0% very difficult. Maternal overweight and obesity were therefore not restricted to economically disadvantaged participants, supporting examination of broader behavioural and structural factors associated with adiposity.

### 3.3. Lifestyle and Dietary Patterns

Table 6 presents mean responses to the Physical Inactivity and Sedentary Lifestyle Scale and the UEBs Scale. Within Scale 3, the highest mean was observed for prolonged mobile phone use (3.42 ± 0.88), closely followed by long television sitting time (3.39 ± 0.90), indicating substantial sedentary screen exposure. Avoidance of planned light activity (3.26 ± 0.91) and fatigue-related inactivity (3.31 ± 0.90) were also elevated. Lower means were recorded for household tasks in a seated manner (2.85 ± 0.92) and extended daytime reclining (2.91 ± 0.95).

Within Scale 4, insufficient fruit and vegetable intake (<5 portions) demonstrated the highest mean (3.51 ± 0.87), followed by large portion sizes (3.46 ± 0.89). Sugar-sweetened beverage intake (3.29 ± 0.92) and fast-food consumption (3.22 ± 0.90) were moderately elevated, whereas breakfast skipping showed the lowest mean (2.73 ± 0.94). These lifestyle and dietary patterns are considered alongside the anthropometric findings presented in Table 7A–D.

### 3.4. Maternal Anthropometric Profile and BMI-Defined Weight Status

Table 7A presents continuous anthropometric measurements in the recruited public antenatal clinic sample (*n* = 3000). The mean BMI was 30.62 ± 6.10 kg/m^2^, while mean WC was 93.80 ± 11.20 cm. Mean BIA-derived VFR was 10.40 ± 3.30 and was interpreted as a continuous pregnancy-period adiposity indicator. These indicators describe maternal adiposity at antenatal assessment and should not be interpreted as pre-pregnancy adiposity or gestational weight gain.

**Figure 3:**
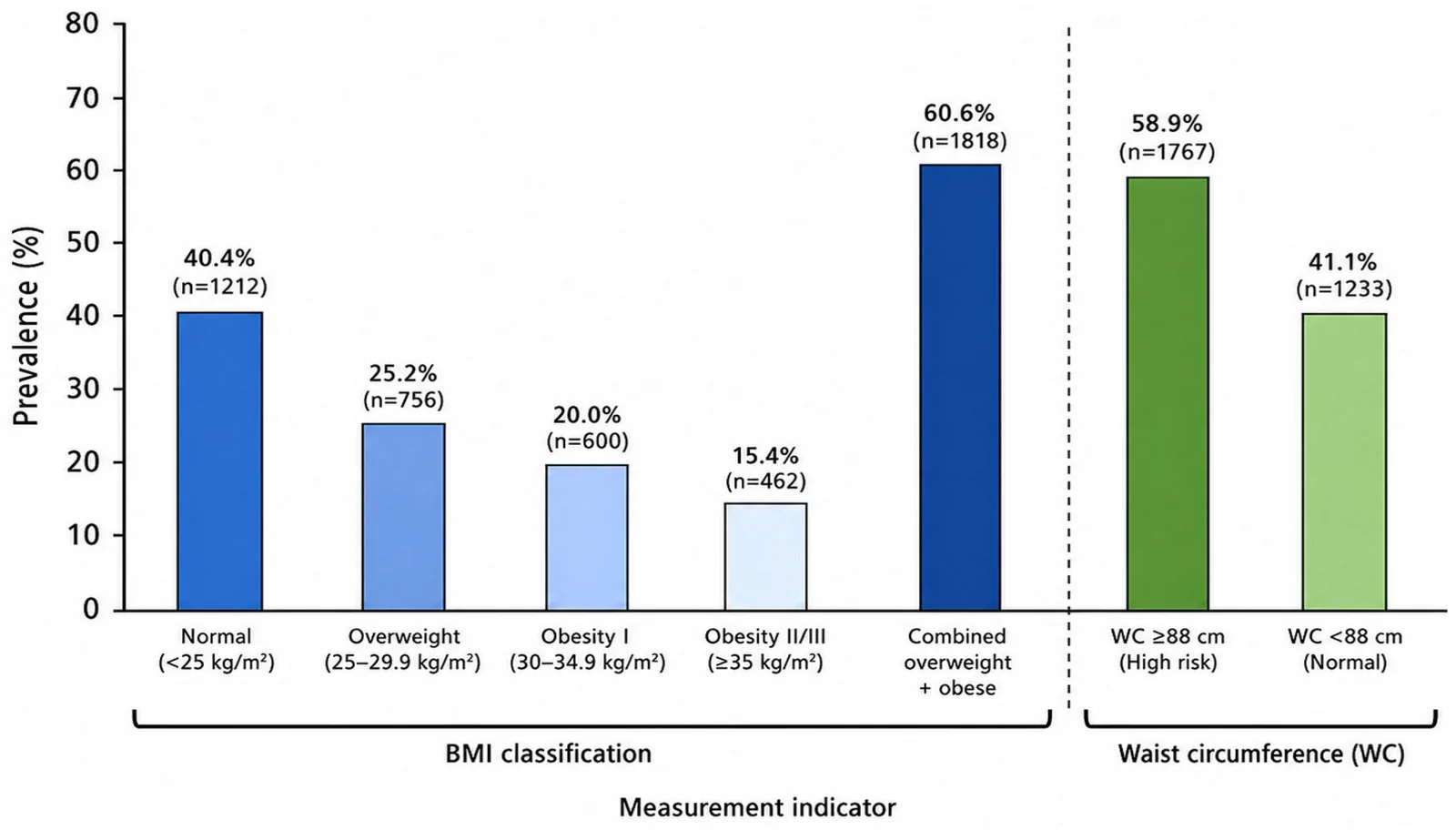
Prevalence of BMI-defined overweight/obesity, elevated WC among women attending participating public antenatal clinics in six Eastern Libyan cities.

Table 7B presents BMI-defined weight status and the prespecified WC indicator. BMI-defined overweight was present in 756 women (25.20%; 95% CI 23.68–26.78), Class I obesity in 600 (20.00%; 95% CI 18.61–21.47), and Class II/III obesity in 462 (15.40%; 95% CI 14.15–16.74). Among the recruited antenatal clinic sample, 1818 women had BMI-defined overweight or obesity, corresponding to a prevalence of 60.60% (95% CI 58.84–62.33). Using the prespecified descriptive threshold, WC ≥ 88 cm was observed in 1767 women (58.90%; 95% CI 57.13–60.65). This threshold was used for epidemiological description and was not treated as a validated pregnancy-specific diagnostic cut-off [12,13,73,74]. Accordingly, WC findings were interpreted alongside continuous values and with particular caution in later gestation (Figure 3).

Values represent adiposity indicators measured at antenatal assessment. WHO BMI categories were applied descriptively, while WC ≥ 88 cm was used as an adult epidemiological threshold and not as a validated pregnancy-specific diagnostic cut-off [37,38,39].

Values represent adiposity indicators measured at antenatal assessment. WHO BMI categories were applied descriptively, while WC ≥ 88 cm and BIA-derived VFR ≥ 10 were treated as prespecified epidemiological thresholds rather than validated pregnancy-specific diagnostic cut-offs.

#### 3.4.1. Maternal Adiposity by Gestational Trimester

Maternal adiposity varied across gestational stages. BMI-defined overweight/obesity increased from 50.00% (95% CI 46.20–53.80) in the first trimester to 58.94% (56.17–61.66) in the second and 68.74% (65.95–71.40) in the third trimester. Elevated WC showed a similar pattern, increasing from 45.45% to 56.91% and 69.10%, respectively (Table 7C). These estimates describe cross-sectional differences between women assessed at different gestational stages and should not be interpreted as within-woman longitudinal change.

#### 3.4.2. Maternal Adiposity by Study City

Geographical variation was observed across the six participating cities. BMI-defined overweight/obesity was highest in Benghazi (65.00%; 95% CI 60.72–69.05), followed by Derna (63.00%), Al Bayda (61.60%), Tobruk (60.00%), Al Marj (58.00%), and Ajdabiya (56.00%). The same descending pattern was observed for elevated WC, ranging from 63.20% in Benghazi to 54.20% in Ajdabiya (Table 7D). These city-specific estimates demonstrate geographical heterogeneity within the recruited multicity antenatal sample and should be interpreted descriptively rather than as evidence of geographical causation.

### 3.5. Hypothesis Testing Results

Hypotheses were tested using adjusted multivariable linear regression for continuous maternal adiposity indicators (BMI, WC, and BIA-derived VFR) and modified Poisson regression with robust variance for BMI-defined overweight/obesity (BMI ≥ 25 kg/m^2^). Models incorporated prespecified covariates, with additional socioeconomic adjustment for H2 and H3. Across H1–H3, adjusted R^2^ values ranged from 0.064 to 0.176, indicating limited-to-modest explanatory power of the full models. All estimates were interpreted as associations rather than causal effects, consistent with the cross-sectional study design.

#### 3.5.1. Hypothesis 1

**H1.**  *Socioeconomic status is associated with maternal adiposity among women attending participating public antenatal clinics in six Eastern Libyan cities.*

The full adjusted H1 models, including socioeconomic indicators and the prespecified covariates, had adjusted R^2^ values of 0.087 for BMI, 0.079 for WC, and 0.064 for VFR. These values represent the explanatory power of the complete adjusted models rather than variance attributable solely to socioeconomic factors. Lower educational attainment was associated with BMI (β = −0.18, *p* < 0.001), WC (β = −0.16, *p* < 0.001), VFR (β = −0.14, *p* = 0.002), and a higher prevalence of BMI-defined overweight/obesity (aPR = 1.24, 95% CI 1.15–1.35, *p* < 0.001). Lower household income was also associated with BMI (β = −0.12, *p* = 0.004), WC (β = −0.11, *p* = 0.006), and overweight/obesity (aPR = 1.18, 95% CI 1.09–1.27, *p* = 0.004). Employment status was not significantly associated with the adiposity outcomes (aPR = 1.03, 95% CI 0.96–1.11, *p* = 0.231). Detailed adjusted associations are presented in Table 8.

#### 3.5.2. Hypothesis 2

**H2.**  *Physical inactivity and sedentary behaviour are associated with maternal adiposity among women attending participating public antenatal clinics in six Eastern Libyan cities.*

In the primary scale-level analysis, the total Physical Inactivity and Sedentary Lifestyle composite score was positively associated with maternal adiposity. Higher composite scores were associated with BMI (β = 0.30, *p* < 0.001), WC (β = 0.27, *p* < 0.001), and BIA-derived VFR (β = 0.23, *p* < 0.001). Each one-point increase in the composite score was associated with a 4% higher prevalence of BMI-defined overweight/obesity (aPR = 1.04, 95% CI 1.03–1.05, *p* < 0.001). These composite-score models constituted the primary test of H2 because the total score represented the psychometrically evaluated construct.

The full adjusted H2 models, including the Physical Inactivity and Sedentary Lifestyle exposure and the prespecified covariates, had adjusted R^2^ values of 0.148 for BMI, 0.132 for WC, and 0.109 for VFR. These values represent the overall explanatory power of the complete models and should not be attributed solely to physical inactivity or sedentary behaviour. Secondary item-level analyses were undertaken to identify the specific behaviours contributing to the overall scale-level association. Prolonged mobile phone use showed the strongest association with BMI-defined overweight/obesity (aPR = 1.30, 95% CI 1.20–1.41, *p* < 0.001), followed by long television sitting (aPR = 1.25, 95% CI 1.16–1.35, *p* < 0.001), avoidance of planned light activity (aPR = 1.22, 95% CI 1.14–1.31, *p* < 0.001), and fatigue or lack of motivation (aPR = 1.21, 95% CI 1.13–1.30, *p* < 0.001). Seated household tasks and extended daytime reclining were not significantly associated with overweight/obesity. Detailed adjusted associations are presented in Table 9.

#### 3.5.3. Hypothesis 3

**H3.**  *Unhealthy eating behaviours are associated with maternal adiposity among women attending participating public antenatal clinics in six Eastern Libyan cities.*

In the primary scale-level analysis, the total UEBs composite score was positively associated with maternal adiposity. Higher UEBs scores were associated with BMI (β = 0.34, *p* < 0.001), WC (β = 0.31, *p* < 0.001), and BIA-derived VFR (β = 0.27, *p* < 0.001). Each one-point increase in the UEBs composite score was associated with a 7% higher prevalence of BMI-defined overweight/obesity (aPR = 1.07, 95% CI 1.05–1.09, *p* < 0.001). The composite-score model constituted the primary test of H3 because it represented the psychometrically evaluated overall unhealthy eating behaviour construct.

The full adjusted H3 models, including the UEBs exposure and the prespecified covariates, had adjusted R^2^ values of 0.176 for BMI, 0.159 for WC, and 0.127 for VFR. These values represent the overall explanatory power of the complete models and should not be attributed solely to unhealthy eating behaviours. Secondary item-level analyses were conducted to determine which dietary behaviours contributed most strongly to the overall scale-level association. Fewer than five fruit and vegetable portions showed the strongest association with BMI-defined overweight/obesity (aPR = 1.36, 95% CI 1.24–1.49, *p* = 0.003), followed by sugar-sweetened beverages (aPR = 1.27, 95% CI 1.16–1.38, *p* = 0.007), fast-food intake (aPR = 1.24, 95% CI 1.14–1.35, *p* = 0.006), and breakfast skipping (aPR = 1.14, 95% CI 1.05–1.24, *p* = 0.011). Large portion size was not significantly associated with overweight/obesity (aPR = 1.05, 95% CI 0.96–1.15, *p* = 0.184). Detailed adjusted associations are presented in Table 10.

The FDR sensitivity analysis did not materially alter inference for BMI-defined overweight/obesity; all associations significant at the nominal *p* < 0.05 level remained significant after Benjamini–Hochberg adjustment (largest FDR-adjusted q = 0.032). No significant trimester interactions were observed for the principal socioeconomic, lifestyle, or dietary exposures (all *p* > 0.05; Table 11).

#### 3.5.4. Gestational-Trimester Interaction and Sensitivity Analyses

Exploratory interaction analyses provided no strong evidence that gestational trimester modified the principal associations between socioeconomic, physical inactivity/sedentary, or dietary exposures and maternal adiposity (all interaction *p*-values > 0.05). Interaction *p*-values for lower educational attainment, prolonged mobile phone use, and low fruit and vegetable intake were 0.312, 0.184, and 0.218, respectively, with similar patterns for the remaining predictors. These findings provided no evidence of substantial heterogeneity in the principal associations across gestational stages (Table 11). First-trimester sensitivity analyses (*n* = 660) produced associations broadly consistent with the full-sample models. Lower educational attainment (aPR = 1.20, 95% CI 1.08–1.34), prolonged mobile phone use (aPR = 1.25, 95% CI 1.10–1.42), and fewer than five fruit and vegetable portions (aPR = 1.30, 95% CI 1.12–1.50) remained the strongest associations within H1, H2, and H3, respectively. The consistency of direction and magnitude in the first-trimester analyses supports the robustness of the principal findings to differences in gestational stage.

## 4. Discussion

The main aim of this study was to characterise maternal adiposity and the proportion of pregnant women meeting standard WHO BMI-defined overweight/obesity thresholds at antenatal assessment in Eastern Libya. The study also examined associations between maternal adiposity and socioeconomic characteristics, unhealthy eating behaviours, physical inactivity, and sedentary behaviour. A cross-sectional design with a multistage, city-stratified sampling strategy across six cities provided broad multicity coverage and improved measurement precision through direct anthropometric assessment rather than self-report [19,22,26]. Participants were largely urban, married, multiparous women in mid-pregnancy with moderate-to-high educational attainment and relatively stable household income. Responses to lifestyle and dietary scales suggested sedentary exposure, particularly screen based behaviours. Suboptimal dietary patterns were also observed, including limited fruit and vegetable intake and larger portion sizes. Similar to global estimates demonstrating a rising burden of maternal overweight in middle income contexts [101], the observed pattern aligns with previous Libyan adult evidence reporting elevated obesity among women prevalence [19,22,25,51]. However, this study extends earlier Libyan pregnancy research through its larger multicity sample and simultaneous assessment of socioeconomic, dietary, and sedentary behaviours across three adiposity indicators. Previous studies were smaller and focused mainly on BMI and pregnancy or delivery outcomes [102,103]. Thus, the principal contribution is broader regional evidence rather than conceptual novelty. Within the Social Ecological Model, the observed findings can be interpreted across interconnected levels, with education and household income representing broader structural and contextual influences and dietary behaviour more proximal behavioural correlates, physical inactivity, and sedentary behaviour representing more proximal behavioural factors. The model is therefore used to organise interpretation of the observed associations rather than to imply mediation or causal pathways that were not tested. Given the cross-sectional design, the observed relationships should not be interpreted causally, as temporal ordering between behaviours and adiposity cannot be established. Residual confounding may also persist despite adjustment for age, parity, and gestational stage, particularly from unmeasured pre-pregnancy adiposity, energy intake, psychosocial factors, clinical conditions, and household environment. BMI, WC, and VFR should therefore be interpreted as pregnancy adiposity indicators because gestational physiological changes may influence their measurement. This distinction is particularly important because gestational changes in maternal tissues and fluid compartments alter body composition, meaning that anthropometric values obtained during pregnancy are not directly equivalent to pre-pregnancy adiposity measures [104].

The study found a high prevalence of maternal adiposity, with more than three-fifths classified as having BMI-defined overweight or obesity, while substantial proportions had elevated WC and BIA-derived VFR. The concordance across these indicators is relevant because BMI does not fully characterise body-fat distribution, whereas WC and BIA-derived VFR may provide complementary epidemiological information during pregnancy [13]. A substantial proportion exceeded the prespecified WC and VFR thresholds; however, these classifications should not be interpreted as diagnostic evidence of visceral adiposity. National adult data report that approximately three-quarters of Libyan adults are living with overweight or obesity, with markedly higher prevalence among women [19,25,51]. Pregnancy-specific Libyan evidence also indicates a substantial but variable burden. Arebi and Aljerbi [105] reported 41% overweight and 23% obesity, while Elghazal et al. [102] identified obesity in 133 of 415 pregnant women. Lower obesity prevalences were reported in Al-Zawia (18.0%) and Tripoli (13.1%) by Alwan et al. [103]. In Saudi Arabia, AlAnnaz et al. [106] reported 40.5% obesity among 8426 women. These estimates support a substantial regional burden but are not directly comparable with the present 60.6% proportion meeting BMI-defined overweight/obesity thresholds at antenatal assessment because recruitment settings, gestational timing, populations, and adiposity definitions differed across studies. The present findings are also consistent with Libya’s broader obesity burden and ongoing socioeconomic, dietary, and activity transitions [19,22,26]. However, the higher prevalence observed in Eastern Libya should not be interpreted as greater underlying susceptibility, as methodological and population differences may partly explain variation between studies.

The maternal profile observed in the present sample therefore reflects broader national patterns where obesity prevalence has increased during recent decades, possibly associated with socioeconomic transition, urbanisation, dietary westernisation, and reduced occupational physical activity [19,22,26]. Similar to international analyses, maternal overweight and obesity have increased across both middle and high-income settings [101,107]. Global modelling research has linked caloric supply, urbanisation, and declining physical labour with maternal adiposity [101]. However, compared with some European cohorts where pre pregnancy BMI has remained relatively stable across shorter intervals [108], the magnitude observed in Libya may reflect contextual pressures including constrained public health programmes and limited preventive initiatives.

Interpretation of the observed adiposity patterns requires careful consideration of the physiological changes accompanying pregnancy. BMI measured during pregnancy reflects maternal anthropometric status at the time of antenatal assessment and is influenced by normal gestational weight gain rather than representing pre-pregnancy adiposity. Consequently, BMI was interpreted as a cross-sectional indicator of maternal adiposity during pregnancy rather than a surrogate for pre-pregnancy BMI. Although pre-pregnancy BMI remains the preferred indicator for obstetric risk assessment, BMI measured during pregnancy is widely used in epidemiological pregnancy research when pre-pregnancy measurements are unavailable, particularly when interpreted alongside gestational age and complementary adiposity measures. Pregnancy-related changes in maternal tissues and body water affect body composition; therefore, BMI, WC and VFR provide complementary rather than exact measures of adiposity during pregnancy [104]. No single measure fully captures maternal adiposity; WC may provide additional information on fat distribution, although methodological heterogeneity limits its individual predictive value [13,104]. Particular caution applies to BIA-derived VFR because impedance estimates depend on tissue conductivity and body-water distribution, both of which change across gestation; VFR should therefore be interpreted as a device-derived pregnancy adiposity indicator rather than a direct anatomical measurement of BIA-derived VFR [109].

Regional evidence highlights this distinction. In Saudi Arabia, Wahabi et al. [110] reported pre-pregnancy overweight and obesity prevalences of 32.2% and 25.8%, respectively, while only 26.3% of women achieved recommended gestational weight gain. However, direct comparison is limited because their study assessed self-reported pre-pregnancy BMI and gestational weight gain, whereas the present study measured BMI, WC and VFR at antenatal enrolment. Verified pre-pregnancy BMI and serial maternal weight measurements were unavailable. Therefore, individual gestational weight gain could not be calculated or included in the analyses. Adjustment for gestational stage reduced, but could not eliminate, the influence of physiological pregnancy-related weight gain on BMI measured at enrolment. Accordingly, BMI at enrolment may reflect both pre-existing maternal adiposity and weight accrued during the current pregnancy. Gestational stage was therefore adjusted for in all models; however, repeated weights were unavailable, preventing assessment of gestational weight gain. Thus, the reported 60.6% represents the proportion of women meeting standard WHO BMI cut-offs for overweight/obesity at antenatal assessment; it should not be interpreted as an estimate of pre-pregnancy overweight/obesity prevalence [37,77]. Given the high prevalence of overweight and obesity (60.6%) in this population, associations with the binary overweight/obesity outcome were estimated using adjusted prevalence ratios rather than odds ratios, providing more appropriate and directly interpretable measures of association for this cross-sectional study. Libyan and MENA evidence links higher maternal BMI with pregnancy-induced hypertension, operative delivery and caesarean delivery; however, these comparisons are contextual because the present study did not assess pregnancy outcomes or causality [102,111].

The findings indicate socioeconomic variation in maternal adiposity in Eastern Libya. Lower educational attainment and household income were associated with a higher prevalence of overweight and obesity, with broadly consistent patterns across BMI, WC, and VFR, whereas employment status was not significantly associated. Evidence from pregnancy cohorts has linked socioeconomic disadvantage with less favourable maternal weight patterns, potentially reflecting differences in health literacy, dietary affordability, and engagement with antenatal care [112,113]. More broadly, socioeconomic gradients in adiposity have been documented across high-income and transitional settings, although their direction and magnitude may vary with economic development and gender norms [19,30,114,115]. Regional evidence is also mixed. Wahabi et al. [110] found no significant association between nutritional scores and the socioeconomic characteristics examined among Saudi pregnant women, contrasting with the associations between lower education, household income, and adiposity observed here. This difference may reflect variation in outcomes, dietary assessment, socioeconomic context, or population characteristics, indicating that socioeconomic relationships with maternal nutrition and adiposity may not be uniform across Middle Eastern populations. Libyan evidence similarly places obesity within a broader context of socioeconomic and nutritional transition [19,25,50]. The weaker association with employment status may indicate that formal employment alone does not adequately capture differences in lifestyle or dietary exposure during pregnancy [116,117,118]. Residual confounding remains possible because education and income may also represent unmeasured differences in food security, health literacy, neighbourhood environment, family circumstances, and healthcare access. Moreover, the cross-sectional design prevents temporal ordering, and reverse causation cannot be excluded; existing adiposity may itself influence mobility, employment, healthcare use, or reported behaviours. Socioeconomic characteristics may therefore assist antenatal risk identification and targeted nutritional support, but these associations should not be interpreted as evidence that socioeconomic disadvantage causes maternal adiposity.

The present results suggest that the observed associations may reflect intersecting contextual influences rather than a single economic pathway. Education and income may shape dietary knowledge, household food purchasing decisions, and perceived ability to manage gestational weight. Qualitative evidence from Libyan adults has described how cultural norms, food availability, and sedentary patterns interact with socioeconomic position to influence obesity risk [25,26,51]. Evidence from pregnant women in Tobruk has also reported socioeconomic influences on nutritional supplementation behaviour during pregnancy [50,119]. International qualitative research reports that women from lower socioeconomic backgrounds may experience barriers to weight management, including restricted access to affordable healthy foods and limited time for self-care [120,121,122]. In contrast, inverse socioeconomic gradients have been reported in some developed settings, particularly among women [123,124,125], suggesting that cultural perceptions of body size and public health messaging may influence these relationships. The observed associations between educational attainment, household income, and adiposity outcomes indicate that these variables may function as measurable socioeconomic correlates within antenatal assessment frameworks. Integration of socioeconomic indicators into routine antenatal assessment protocols may facilitate structured identification of populations requiring targeted nutritional interventions.

The study findings showed that lower engagement in planned light activity, minimal daily movement, and motivational fatigue were associated with a higher prevalence of BMI-defined overweight and obesity. Similar associations were observed across BMI, WC and VFR, supporting the consistency of the findings across different adiposity measures. Such convergence strengthens the internal consistency of the observed associations but should not be interpreted as validation of any individual measure, particularly because pregnancy-related physiological changes affect these adiposity indicators differently [104]. Earlier Libyan evidence reported an inverse relationship between total physical activity across work, transport, and recreational domains and obesity, particularly among women [22,25,26]. That research indicated that total physical activity remained negatively associated with BMI in women even after adjustment for confounding factors. Structural and sociocultural conditions in Libya, including the availability of gender-segregated recreational facilities and evolving beauty norms influenced by Western media, have also been linked with patterns of female physical activity participation [22,25,126]. Persistent barriers may limit sustained engagement in physical activity [26,127,128]. These barriers include unsafe environments during political instability, long travel distances to facilities, extreme summer temperatures, reliance on cars, and time constraints [26,90]. Evidence from systematic synthesis indicates that correlates of physical activity are more consistently identified than those related to sedentary behaviour. Individual and psychosocial determinants show clearer directional relationships, whereas sedentary exposures often show heterogeneous or inconsistent patterns [16,129,130]. The observed associations align with the relevance of incorporating feasible, low-intensity physical activity guidance within antenatal care frameworks. Walking-based recommendations adapted to environmental and sociocultural conditions may represent an appropriate approach within routine maternal health services. The observed associations indicate behavioural correlates of adiposity but do not establish whether lower physical activity preceded or resulted from increased adiposity during pregnancy.

The results further showed that screen-based sedentary behaviours, particularly prolonged mobile phone use and television viewing, were associated with a higher prevalence of BMI-defined overweight and obesity. These associations were observed using adjusted prevalence ratios derived from modified Poisson regression and remained consistent across the continuous adiposity measures. Previous Libyan findings reported a positive relationship between sedentary behaviour and BMI among women [19,22,26]. Several mechanisms have been proposed including prolonged television viewing, exposure to food advertising, eating during screen use, and reduced metabolic rate during prolonged sitting [26,131,132]. Within Libyan households, employment of domestic workers and reliance on labour-saving appliances may further reduce routine physical effort among women [22,25,26]. Unlike women, men have been reported to combine sedentary and physically active activities within the same day, which may moderate the metabolic effects of prolonged sitting [19,25,26]. Current evidence suggests that associations between sedentary behaviour and health outcomes during pregnancy are not uniformly consistent. Variability across study designs, measurement methods, and outcome definitions indicates that interpretation should remain cautious [17,18]. Furthermore, systematic review findings highlight that sedentary behaviour correlates often demonstrate limited consistency across studies, with a substantial proportion of investigated factors showing indeterminate or non-significant associations, thereby restricting causal inference [16,17]. Observed associations in the present study should therefore be interpreted as indicative rather than definitive. This caution reflects the cross-sectional design, potential residual confounding, and the complex interaction between sedentary patterns and overall physical activity behaviours [16,17,107]. However, the magnitude of these associations remains subject to measurement-related limitations, as sedentary behaviours were assessed using self-reported instruments that may introduce recall error and social desirability bias. Consequently, the observed associations represent behavioural correlates within a cross-sectional framework, and confirmation through longitudinal designs is required to establish temporal consistency and strengthen causal inference. Interpretation should therefore remain cautious, recognising that sedentary behaviour and adiposity may influence one another through complex and potentially bidirectional pathways. In particular, greater existing adiposity may itself reduce mobility and increase sitting or screen-based activity, including mobile phone use; therefore, the direction of these associations cannot be established from the present cross-sectional data.

The study findings showed that unhealthy eating behaviours were associated with a higher prevalence of BMI-defined overweight and obesity among pregnant women. Similar associations were observed for continuous BMI, WC, and VFR. Inadequate fruit and vegetable intake showed the strongest association. Sugar-sweetened beverage consumption, fast-food intake, and breakfast skipping were also associated with higher adiposity. These patterns were broadly consistent across BMI, WC, and VFR. Consistency across the three indicators suggests that the dietary associations were not confined to BMI alone; however, WC and particularly BIA-derived VFR should remain complementary pregnancy adiposity indicators rather than definitive measures of maternal fat distribution [13,104]. Previous Libyan adult studies similarly reported associations between obesity, fast-food consumption, sugar-sweetened beverages, and breakfast skipping [22,25,26]. Similar associations with unhealthy eating behaviour, physical inactivity, and sedentary lifestyle have recently been reported among Libyan children and adolescents [133,134]. However, inadequate fruit and vegetable intake was less prominent in earlier adult research. Regional evidence provides both support and contrast. Wahabi et al. [110] reported suboptimal dietary practices among Saudi pregnant women. Only 62.3% reported adequate fruit and vegetable intake, while 23.9% reported adequate fish intake. However, their nutritional risk and diet-quality scores were not significantly associated with gestational weight gain. This differs partly from the present findings, where individual dietary behaviours were associated with maternal adiposity measured during pregnancy. The difference may reflect variation in outcomes and assessment methods. Wahabi et al. [110] examined gestational weight gain using a FIGO-based nutritional assessment, whereas the present study examined BMI, WC, and VFR in relation to specific dietary behaviours. Differences in sampling and population characteristics may also contribute to the different findings. Dietary behaviours may therefore show different relationships with maternal weight indicators across regional populations. Evidence from pregnant populations has also associated energy-dense dietary patterns with maternal weight and metabolic outcomes [135,136,137]. Behavioural influences on unhealthy dietary practices during pregnancy have been reported [31,138]. Nevertheless, the cross-sectional design does not establish the temporal direction of these associations. Women with greater adiposity may have changed their dietary behaviour following antenatal counselling or concerns about their weight. Reverse causation therefore remains possible. Self-reported dietary behaviours may also be affected by recall and social desirability bias. Residual confounding may remain because total energy intake, pre-pregnancy diet, nausea, gestational diabetes counselling, and food availability were not fully measured. The findings may support antenatal nutritional counselling that considers dietary quality, micronutrient intake, and regular meal patterns rather than caloric intake alone. Recent Libyan intervention evidence also supports the relevance of structured diet and lifestyle modification in clinical settings [133]. Accordingly, the dietary findings should be interpreted as associations with maternal adiposity during pregnancy rather than evidence that these behaviours caused overweight or obesity.

The present results further show that large portion sizes did not demonstrate an independent association with maternal adiposity outcomes. Frequency and quality of dietary intake showed clearer relationships. Earlier Libyan adult research identified portion size as a dominant predictor of obesity [22,26], suggesting differences between general and gestational populations. Pregnancy-related appetite regulation, social eating norms, or increased nutritional awareness may influence portion-related behaviour once other dietary patterns are considered [139,140]. Associations observed for fast-food and sugar-sweetened beverages correspond with international evidence linking ultra-processed foods and liquid sugars with adiposity through low satiety and high glycaemic load mechanisms [141,142,143]. Breakfast skipping has also been associated with altered metabolic regulation and compensatory intake later in the day within both adult and maternal research [144,145,146]. Measurement of portion size relied on self-reported data rather than direct quantification. This approach may introduce reporting variability and limit precision of estimation. Interpretation of BMI, WC, and VFR should also remain cautious because pregnancy-related physiological changes may influence adiposity measures. This limitation is especially relevant to VFR because gestational expansion and redistribution of body water can alter impedance-derived body-composition estimates independently of changes in adipose tissue [109]. Furthermore, the cross-sectional design identifies associations rather than causal relationships between dietary behaviours and maternal adiposity.

## 5. Theoretical and Practical Implications

The study situates maternal adiposity in Eastern Libya within a social determinants perspective rather than attributing gestational weight patterns solely to individual behaviour. The Social Ecological Model provides a framework for interpreting these findings across different but interacting levels of influence. Lower educational attainment and household income represent broader structural and contextual conditions, whereas sedentary exposure, physical inactivity, and dietary behaviours represent more proximal behavioural correlates. Associations between lower educational attainment, lower household income, sedentary exposure, and dietary quality suggest that structural conditions may influence opportunities for physical activity, food access, and health literacy during pregnancy [38,121]. Evidence from Libya linking political instability, reliance on motorised transport, and home-based routines with obesity risk indicates that environmental context may shape behavioural patterns [19,25,147]. Similar to regional analyses describing barriers to physical activity across Middle East and North Africa settings, inactivity during pregnancy may reflect environmental limitations alongside individual behaviour [148,149]. Accordingly, the coexistence of socioeconomic and behavioural associations suggests that maternal adiposity during pregnancy may be related to both individual behaviours and the wider circumstances in which those behaviours occur. However, the present cross-sectional analysis did not test mediation, interaction, or pathways between ecological levels; therefore, the Social Ecological Model should be interpreted as an organising framework rather than a tested causal mechanism.

Study findings have practical implications for maternal health planning. The high proportion of BMI-defined overweight and obesity, together with elevated WC and BIA-derived VFR, supports routine antenatal identification of women with elevated adiposity. aPR analyses identified associations between socioeconomic characteristics, physical inactivity, sedentary behaviour, dietary behaviours, and BMI-defined overweight/obesity, which may inform antenatal assessment and the development of future prevention strategies without implying that modification of these factors would necessarily reduce adiposity. Integration of routine adiposity screening, including BMI and central indicators, within antenatal protocols may support earlier identification of women experiencing elevated risk, particularly among those with lower educational attainment or lower household income. Policy initiatives addressing maternal nutrition and physical activity could consider urban infrastructure realities, including limited recreational spaces and reliance on motorised transport. Support for feasible home-based movement and improved access to fruit and vegetables through subsidy or supply systems may represent practical responses within the Libyan context. Clinical professionals, including gynaecologists and midwives, may incorporate brief counselling addressing screen exposure, dietary quality, and gestational weight monitoring during routine antenatal consultations. Primary healthcare providers may benefit from training that integrates socioeconomic assessment with behavioural advice, while evaluation of culturally adapted antenatal lifestyle programmes may clarify feasible prevention strategies within maternal healthcare services. Recent prospective evidence from Tobruk has also evaluated dietary management within Libyan chronic-disease care [150].

## 6. Strengths and Limitations

Several methodological features strengthen interpretation of the findings. Recruitment of 3000 pregnant women across six cities in Eastern Libya provided wide geographic coverage and improved statistical precision. Direct anthropometric assessment using standardised equipment provided objectively measured BMI together with complementary WC and BIA-derived VFR indicators, avoiding reliance on self-reported anthropometric measurements. A multistage city-stratified design with systematic random recruitment within participating facilities provided broad multicity coverage; however, facility selection was eligibility- and feasibility-based rather than probability-based. Psychometric evaluation of the Arabic questionnaire, including validity and reliability assessment, supported measurement consistency across behavioural and socioeconomic variables. Statistical adjustment for key demographic and obstetric factors further strengthened analytical stability.

Interpretation of the results should also consider several constraints. The cross-sectional design measured exposures and adiposity indicators at a single time point, limiting temporal interpretation because behavioural changes during pregnancy may occur before or after weight gain [33]. Cross-sectional studies are useful for estimating prevalence and describing population health patterns, but simultaneous measurement of exposures and outcomes restricts causal interpretation and makes temporal ordering uncertain [33,34]. Accordingly, the present findings should be interpreted as associations between socioeconomic, dietary, lifestyle, and adiposity indicators, rather than evidence that these exposures caused overweight or obesity during pregnancy. Therefore, the observed socioeconomic, dietary, and lifestyle variables should be regarded as correlates rather than risk factors because temporal ordering cannot be established [45,46,47,149]. Although cross-sectional evidence can contribute to epidemiological understanding, causal claims require stronger attention to temporality, reverse causation, exposure timing, and residual bias [151]. Reverse causality is particularly plausible for physical inactivity, sedentary behaviour, mobile phone use, and dietary behaviours, because existing maternal adiposity may itself affect mobility, sitting and screen time, and reported eating patterns.

Recruitment through antenatal facilities may have underrepresented women with irregular service attendance or limited healthcare access. Women attending public clinics may differ from non-attenders in socioeconomic circumstances, health-seeking behaviour, pregnancy risk profile, and healthcare access [152,153]. Although recruitment across multiple facilities in six cities and systematic use of attendance registers and clinic logbooks reduced informal selection, the sampling frame was restricted to public antenatal services [154,155]. Women receiving care exclusively in private facilities or not attending antenatal care were therefore not represented. Consequently, findings are most generalisable to women attending participating public antenatal clinics and should not be anticipated to represent all pregnant women in Eastern Libya, as healthcare-based sampling may limit external validity [45,156].

Sampling procedures also require cautious interpretation. Systematic sampling within facilities relied on stable patient flow, and adjustments during low-attendance sessions may have affected strict sampling randomness. Cluster-based recruitment may have introduced intra-facility correlation, which can increase variance if clustering is not fully addressed during analysis [157,158]. This issue was partly addressed by treating clinic as the primary sampling unit and city as the stratification level during analysis. Nevertheless, residual design effects may remain because women attending the same facility may share similar socioeconomic, geographic, or service-access characteristics. Although equal allocation across the six cities improved comparability, the absence of sampling weights means that estimates should be interpreted as describing the recruited antenatal clinic sample rather than as weighted population estimates for all pregnant women in Eastern Libya [51,52].

Measurement considerations should also be acknowledged. An additional limitation is that verified pre-pregnancy BMI, serial gestational weight measurements, and gestational weight gain data were unavailable for analysis. Consequently, BMI reflected maternal anthropometric status at the time of antenatal assessment rather than adiposity before conception and may have partly incorporated normal physiological gestational weight gain and changes in body composition. Although gestational stage was included as an adjustment variable in all multivariable analyses to minimise potential confounding arising from physiological pregnancy-related weight changes, residual influence cannot be completely excluded. Residual confounding may remain because several important clinical variables were unavailable or inconsistently recorded. These included pre-pregnancy BMI, GWG, smoking, previous gestational diabetes, hypertensive disorders, and other pregnancy complications. Therefore, adjusted estimates should be interpreted as associations rather than causal effects [33,34,35,36,99,100]. Accordingly, the findings should be interpreted as associations with maternal adiposity measured during pregnancy rather than pre-pregnancy BMI or gestational weight gain. For the same reason, classifications based on the standard WHO BMI thresholds were used descriptively and the reported 60.6% should be interpreted as BMI-defined weight status at antenatal assessment rather than an estimate of pre-pregnancy overweight/obesity prevalence. Future prospective studies incorporating objectively measured pre-pregnancy BMI together with repeated anthropometric and gestational weight assessments throughout pregnancy would allow more precise evaluation of maternal adiposity trajectories and their independent relationships with maternal and neonatal outcomes.

Bioelectrical impedance assessment using the TANITA RD-545HR device enabled objective estimation of body composition. Nevertheless, physiological changes during pregnancy, including fluid shifts and uterine enlargement, may influence impedance-derived fat estimates and waist circumference interpretation, particularly during later gestation [12,13]. Plasma volume expansion, changes in total body-water distribution, and progressive uterine growth may alter both impedance-derived estimates and abdominal circumference independently of true changes in visceral adipose tissue, particularly during the second and third trimesters. Although evidence supports early pregnancy waist circumference and early-to-mid pregnancy BIA-derived VFR assessment, interpretation during late pregnancy remains less definitive because anatomical and physiological changes may affect measurement stability across trimesters [44,66]. Recent evidence indicates that bioelectrical impedance-derived body fat percentage and waist circumference may still provide clinically informative associations during the second trimester and middle-to-late pregnancy [43,67]. However, BIA-derived VFR carries additional uncertainty because pregnancy-related changes in hydration and body-water distribution may affect impedance estimates [73,74,109]. Accordingly, VFR should be interpreted as a supportive indicator of pregnancy-period adiposity rather than a precise or diagnostic measure of BIA-derived VFR, particularly during the second and third trimesters. WC ≥ 88 cm was retained as an adult descriptive threshold [78], whereas VFR was analysed continuously because no validated pregnancy-specific cut-off is available [38,39,87]. The first-trimester sensitivity analyses provided a robustness assessment of the observed associations but were not interpreted as validation of these thresholds.

Lifestyle and dietary behaviours were assessed through self-reported Likert-scale responses, which may be influenced by recall error or social desirability bias within maternity settings [51]. Nurse supervision and immediate form checking reduced item-level missingness, although minor missing values cannot be fully excluded in survey-based research. Remaining incomplete values were handled conservatively through predefined coding and complete-case analysis [82,159]. Questionnaire scores should therefore be interpreted as structured indicators of reported behavioural exposure rather than direct measurements of diet, physical activity, or sedentary time. Although composite Likert-type scoring supports statistical analysis and comparison across participants, raw questionnaire scores do not provide interval-level clinical measurement in the same way as anthropometric variables [60,61].

Self-reported behavioural and dietary data are vulnerable to information bias because participants may forget events, misclassify frequency, simplify usual behaviour, or adjust answers towards socially acceptable responses [64,65]. Dietary assessment is particularly sensitive to under-reporting, intake-related bias, and person-specific reporting patterns that can attenuate or distort associations in nutritional epidemiology [160]. To reduce this risk, the questionnaire used short and clearly defined recall periods: the past 7 days for physical inactivity and sedentary behaviour and the past month for UEBs. Closed response categories were also used to improve consistency across participants. Nevertheless, differential recall and reporting bias cannot be excluded, particularly if women with higher adiposity reported diet or activity differently from women with lower adiposity [78]. Behavioural findings should therefore be interpreted as self-reported exposure patterns rather than precise objective measures.

Socioeconomic indicators were operationalised using education, income, and employment status, although these proxies may not fully represent informal economic activity or neighbourhood-level deprivation within the Libyan context. Regression models assumed correct specification and absence of unmeasured confounding; however, observational studies remain vulnerable to incomplete adjustment because some exposures may be unmeasured, measured with error, or grouped into broad categories that do not capture within-group variation [57,90,161]. In this study, pre-pregnancy lifestyle behaviours, household food environment, psychological stress, cultural dietary practices, and neighbourhood-level deprivation were not directly measured. Because overweight and obesity were highly prevalent in the study population, modified Poisson regression with robust variance was used to estimate adjusted prevalence ratios rather than odds ratios for the binary overweight/obesity outcome. This approach provides more directly interpretable measures of association for common outcomes in cross-sectional studies. Nevertheless, the reported adjusted prevalence ratios should be interpreted as measures of association rather than evidence of causality because of the cross-sectional study design. Multiple testing was addressed by prioritising the H2 and H3 composite scores and treating item-level analyses as exploratory. Benjamini–Hochberg FDR adjustment did not materially alter inference; associations significant at the nominal *p* < 0.05 level remained significant after adjustment. However, no omnibus multiplicity correction was applied across the correlated BMI, WC, and VFR outcomes; therefore, secondary associations were interpreted cautiously

Sampling procedures also require cautious interpretation. Systematic sampling within facilities relied on stable patient flow, and adjustments during low-attendance sessions may have affected strict sampling randomness. Cluster-based recruitment may have introduced intra-facility correlation, which can increase variance if clustering is not fully addressed during analysis. This issue was partly addressed by treating clinic as the primary sampling unit and city as the stratification level during analysis. Nevertheless, residual design effects may remain because women attending the same facility may share similar socioeconomic, geographic, or service-access characteristics. Although equal allocation across the six cities improved comparability, the absence of sampling weights means that estimates should be interpreted as representative of the recruited antenatal clinic population rather than as weighted population estimates for all pregnant women in Eastern Libya [51,52].

## 7. Recommendations for Future Studies

Future studies in Libya may benefit from prospective pregnancy cohort designs beginning before conception or during early gestation. Longitudinal follow-up may allow examination of pre-pregnancy BMI, gestational weight trajectories, and repeated adiposity measurements across pregnancy and the postpartum period. Such approaches could reduce distortion associated with advancing gestation when interpreting WC and bioelectrical impedance outcomes [12,13]. Recruitment strategies extending beyond antenatal clinics to community health centres, outreach programmes, and private healthcare facilities may improve representation of women with irregular service utilisation, particularly in peri-urban and rural areas. Analytical approaches incorporating sampling weights, cluster-adjusted variance estimation, and appropriate estimation of prevalence ratios for common binary outcomes may further strengthen epidemiological inference when multistage sampling structures are used [88].

Further studies may incorporate objective measures of physical activity, including accelerometry where feasible, to reduce reporting bias and quantify sedentary exposure during pregnancy more accurately [49]. Mixed-methods approaches may also examine how transport dependence, safety concerns, domestic labour structures, and food availability influence behavioural patterns across socioeconomic groups in Libyan cities. Intervention-focused studies evaluating antenatal programmes combining culturally appropriate nutrition guidance with feasible movement strategies delivered through primary healthcare services may provide additional evidence relevant to maternal health planning. Follow-up beyond delivery may permit examination of maternal and early childhood outcomes associated with gestational adiposity.

## 8. Conclusions

A substantial proportion of women in the recruited public antenatal clinic sample across six Eastern Libyan cities had elevated pregnancy-period adiposity indicators, including BMI-defined weight status, WC, and BIA-derived VFR measured at antenatal assessment. Lower educational attainment and household income, greater physical inactivity and sedentary behaviour, and more frequent unhealthy eating behaviours were associated with higher maternal adiposity, while some individual exposures showed no independent association. These findings provide region-specific evidence on socioeconomic and behavioural correlates of maternal adiposity and may inform antenatal assessment and future preventive research. However, the cross-sectional design does not establish causality or demonstrate that changing these characteristics or behaviours would reduce maternal adiposity. Prospective and intervention studies are required to evaluate these relationships longitudinally.

## Figures and Tables

**Table 1:** Recruitment and response rates across the six participating cities in Eastern Libya (*n* = 3000).

City	Eligible Women Approached	Completed	Response Rate
Tobruk	546	500	91.6%
Derna	540	500	92.6%
Al Marj	529	500	94.5%
Al Bayda	528	500	94.7%
Benghazi	525	500	95.2%
Ajdabiya	524	500	95.4%
Overall	3192	3000	94.0%

**Table 2:** Anthropometric classification and recommended gestational weight gain.

Measurement Domain	Category	Cut-Off/Recommended Range
BMI	Underweight	<18.5 kg/m^2^
	Healthy weight	18.5–24.9 kg/m^2^
	Overweight	25.0–29.9 kg/m^2^
	Obesity	≥30.0 kg/m^2^
Gestational weight gain (singleton pregnancy)	Underweight	12.7–18.1 kg
	Healthy weight	11.3–15.9 kg
	Overweight	6.8–11.3 kg
	Obesity	5.0–9.1 kg
WC	Increased metabolic risk	≥80 cm
	Prespecified descriptive threshold	≥88 cm *
BIA-derived VFR	Continuous device-derived rating	No pregnancy-specific diagnostic cut-off applied ^†^

Assessed in early pregnancy (≤12–14 weeks’ gestation). Sources [9,10,11,13]. * WC ≥ 88 cm was used as an adult epidemiological threshold, not a validated pregnancy-specific diagnostic cut-off [78]. ^†^ Adult manufacturer VFR ranges are 1–12.5 healthy and 13–59 excessive; these were not applied diagnostically in pregnancy [37,44].

**Table 3:** Psychometric properties of the study questionnaire (SPSS v29).

Scale	Items (*n*)	Cohen’s κ	Mean CVR	KMO	Bartlett’s χ^2^ (df)	Variance Explained (%)	Cronbach’s α	Test–Retest ICC (95% CI)
Demographic profile	8	0.82	0.90	—	—	—	—	—
SES	8	0.78	0.88	—	—	—	—	—
Physical inactivity and sedentary lifestyle	12	0.76	0.85	0.87	1485.32 (66) *	59.20	0.86	0.88 (0.82–0.92) ^†^
UEBs	5	0.74	0.82	0.81	612.45 (10) *	57.10	0.79	0.83 (0.75–0.89) ^†^

* *p* < 0.001. ^†^ ICC values are reported with 95% confidence intervals.

**Table 4:** Demographic profile of pregnant participants (*n* = 3000).

Variable	Category	Frequency (%)
Age Group	<20	150 (5%)
	20–24	540 (18%)
	25–29	720 (24%)
	30–34	660 (22%)
	35–39	720 (24%)
	≥40	210 (7%)
Gestational Stage	1st trimester	660 (22%)
	2nd trimester	1230 (41%)
	3rd trimester	1110 (37%)
Marital Status	Married	2880 (96%)
	Divorced/Widowed/Other	120 (4%)
Parity	0	660 (22%)
	1	840 (28%)
	2–3	1020 (34%)
	≥4	480 (16%)
Residence	Urban	1920 (64%)
	Peri-urban	690 (23%)
	Rural	390 (13%)
Pregnancy Type	Singleton	2910 (97%)
	Twins/Multiples	90 (3%)

**Table 5:** Socioeconomic profile of pregnant participants (*n* = 3000).

Variable	Category	Frequency (%)
Education	Preparatory/Basic	200 (6.7%)
	Secondary	600 (20.0%)
	Diploma	210 (7.0%)
	University degree	1810 (60.3%)
	Postgraduate	180 (6.0%)
Employment Status	Employed	1260 (42%)
	Self-employed	540 (18%)
	Homemaker	930 (31%)
	Unemployed	270 (9%)
Income (LYD)	<1000	270 (9%)
	1000–1999	480 (16%)
	2000–2999	990 (33%)
	3000–3999	720 (24%)
	≥4000	540 (18%)
Household Size	1–2	750 (25%)
	3–4	1650 (55.0%)
	5–6	400 (13.3%)
	≥7	200 (6.7%)
Financial Strain	Comfortable	1800 (60%)
	Coping	900 (30%)
	Difficult	240 (8%)
	Very difficult	60 (2%)

**Table 6:** Lifestyle and dietary scale responses (*n* = 3000).

Scale	Item	Mean ± SD
Physical Inactivity	Long mobile phone use	3.42 ± 0.88
	Long TV sitting	3.39 ± 0.90
	Avoided planned activity	3.26 ± 0.91
	Travelled short distances by car	3.20 ± 0.96
	Extended reclining	2.91 ± 0.95
Unhealthy Eating	<5 fruit/vegetable portions	3.51 ± 0.87
	Large portion sizes	3.46 ± 0.89
	Sugar-sweetened beverages	3.29 ± 0.92
	Fast-food intake	3.22 ± 0.90
	Skipping breakfast	2.73 ± 0.94

**Table 7:** (A) Anthropometric measurements for maternal adiposity. (B) BMI-defined weight status and elevated WC and VFR indicators at antenatal assessment (*n* = 3000). (C) Maternal adiposity prevalence by gestational trimester. (D) Maternal adiposity prevalence by city.

**A**							
**Anthropometric Variable**				**Mean ± SD**			
Height (cm)				162.40 ± 6.85			
Weight (kg)				80.10 ± 14.90			
BMI (kg/m^2^)				30.62 ± 6.10			
Body fat (%)				35.20 ± 6.70			
VFR				10.40 ± 3.30			
WC (cm)				93.80 ± 11.20			
**B**							
**Measurement Indicator**			**Category**			***n* (%)**	
BMI classification			Normal (<25 kg/m^2^)			1182 (39.40%)	
			Overweight (25–29.9 kg/m^2^)			756 (25.20%)	
			Class I obesity (30–34.9 kg/m^2^)			600 (20.00%)	
			Class II/III obesity (≥35 kg/m^2^)			462 (15.40%)	
			Combined overweight and obesity			1818 (60.60%)	
WC			≥88 cm, prespecified descriptive threshold			1767 (58.90%)	
			<88 cm, below descriptive threshold			1233 (41.10%)	
**C**							
**Gestational Stage**	***n***	**BMI ≥ 25 kg/m^2^, *n* (%)**	**95% CI**	**WC ≥ 88 cm, *n* (%)**	**95% CI**	**VFR ≥ 10, *n* (%)**	**95% CI**
First-trimester	660	330 (50.00%)	46.20–53.80	300 (45.45%)	41.69–49.27	270 (40.91%)	37.22–44.70
Second-trimester	1230	725 (58.94%)	56.17–61.66	700 (56.91%)	54.13–59.65	610 (49.59%)	46.80–52.38
Third-trimester	1110	763 (68.74%)	65.95–71.40	767 (69.10%)	66.32–71.75	683 (61.53%)	58.63–64.35
Overall	3000	1818 (60.60%)	58.84–62.33	1767 (58.90%)	57.13–60.65	1563 (52.10%)	50.31–53.88
**D**							
**City**	***n***	**BMI ≥ 25 kg/m^2^, *n* (%)**		**95% CI**	**WC ≥ 88 cm, *n* (%)**	**95% CI**	
Benghazi	500	325 (65.00%)		60.72–69.05	316 (63.20%)	58.89–67.31	
Derna	500	315 (63.00%)		58.68–67.12	307 (61.40%)	57.06–65.56	
Al-Bayda	500	308 (61.60%)		57.26–65.76	300 (60.00%)	55.65–64.20	
Tobruk	500	300 (60.00%)		55.65–64.20	292 (58.40%)	54.03–62.64	
Al-Marj	500	290 (58.00%)		53.63–62.25	281 (56.20%)	51.82–60.49	
Ajdabiya	500	280 (56.00%)		51.62–60.29	271 (54.20%)	49.82–58.52	
Overall	3000	1818 (60.60%)		58.84–62.33	1767 (58.90%)	57.13–60.65	

Note: BMI, WC and VFR were measured during pregnancy at study enrolment. WHO BMI thresholds were used descriptively and do not represent pre-pregnancy BMI classification. WC ≥ 88 cm was used as a descriptive epidemiological threshold, not validated pregnancy-specific diagnostic cut-off. The six cities each contributed 500 women in the original study design.

**Table 8:** Adjusted associations between socioeconomic indicators and maternal adiposity outcomes (*n* = 3000).

SES Indicator	r (BMI)	β (BMI)	β (WC)	β (VFR)	Adjusted aPR (95% CI)	*p*-Value
Lower educational attainment	−0.21	−0.18	−0.16	−0.14	1.24 (1.15–1.35)	<0.001
Lower household income	−0.17	−0.12	−0.11	−0.09	1.18 (1.09–1.27)	0.004
Employment status	−0.06	−0.05	−0.04	−0.03	1.03 (0.96–1.11)	0.231

Note: Adjusted for age, parity, and gestational stage. Modified Poisson regression with robust variance for BMI ≥ 25 kg/m^2^. VIF < 2.5; negative coefficients indicate inverse coding where applicable. Complete-case *n* = 3000; no missing observations were excluded.

**Table 9:** Adjusted associations between the Physical Inactivity and Sedentary Lifestyle composite score, individual behavioural indicators, and maternal adiposity outcomes (*n* = 3000).

Analysis	Behaviour/Exposure	r (BMI)	β (BMI)	β (WC)	β (BIA-Derived VFR)	Adjusted aPR (95% CI)	*p*-Value
Primary composite analysis	Total Physical Inactivity and Sedentary Lifestyle score (12–60)	0.34	0.30	0.27	0.23	1.04 (1.03–1.05)	<0.001
Secondary item analysis	Avoided planned light activity	0.27	0.25	0.22	0.19	1.22 (1.14–1.31)	<0.001
	Chose sitting instead of walking	0.24	0.21	0.19	0.17	1.18 (1.10–1.27)	0.003
	Travelled short distances by car	0.22	0.18	0.16	0.14	1.14 (1.06–1.23)	0.012
	Household tasks in seated manner	0.09	0.07	0.05	0.04	1.03 (0.96–1.11)	0.247
	Felt too tired/unmotivated	0.26	0.23	0.21	0.18	1.21 (1.13–1.30)	<0.001
	Very little daily movement	0.25	0.22	0.20	0.17	1.19 (1.11–1.28)	0.002
	Long TV sitting	0.28	0.26	0.23	0.20	1.25 (1.16–1.35)	<0.001
	Long mobile phone use	0.29	0.27	0.24	0.21	1.30 (1.20–1.41)	<0.001
	Long sitting with family	0.18	0.15	0.13	0.11	1.11 (1.02–1.19)	0.021
	Prolonged sitting during travel	0.23	0.20	0.18	0.15	1.17 (1.08–1.26)	0.007
	Eating while using screen	0.20	0.17	0.15	0.13	1.12 (1.04–1.21)	0.026
	Extended daytime reclining	0.11	0.09	0.08	0.06	1.05 (0.98–1.13)	0.173

Note: Primary H2 exposure: total composite score; individual items were secondary analyses. Adjustment: age, parity, gestational stage and SES. Overweight/obesity: modified Poisson regression with robust variance (BMI ≥ 25 kg/m^2^). Abbreviations: aPR, adjusted prevalence ratio; WC, waist circumference; VFR, BIA-derived visceral fat rating. Diagnostics: VIF < 2.5. Complete-case analysis: *n* = 3000; no missing observations.

**Table 10:** Adjusted associations between the UEBs composite score, individual dietary behaviours, and maternal adiposity outcomes (*n* = 3000).

Analysis	Eating Behaviour/Exposure	β (BMI)	β (WC)	β (BIA-Derived VFR)	Adjusted aPR (95% CI)	*p*-Value
Primary composite analysis	Total UEBs score (5–25)	0.34	0.31	0.27	1.07 (1.05–1.09)	<0.001
Secondary item analysis	Fast-food intake	0.24	0.22	0.19	1.24 (1.14–1.35)	0.006
	Sugar-sweetened beverages	0.26	0.24	0.21	1.27 (1.16–1.38)	0.007
	<5 fruit/vegetable portions	0.32	0.29	0.25	1.36 (1.24–1.49)	0.003
	Skipping breakfast	0.19	0.17	0.15	1.14 (1.05–1.24)	0.011
	Large portion sizes	0.07	0.06	0.05	1.05 (0.96–1.15)	0.184

Note: Primary H3 exposure: total UEBs composite score; individual items: secondary analyses. Adjusted for age, parity, gestational stage and SES. Modified Poisson regression with robust variance for BMI ≥ 25 kg/m^2^. aPR = adjusted prevalence ratio; WC = waist circumference; VFR = BIA-derived VFR. VIF < 2.5. Complete-case *n* = 3000; missing data = 0.

**Table 11:** First-trimester sensitivity analyses and gestational-trimester interactions for principal exposures associated with BMI-defined overweight/obesity.

Hypothesis	Exposure	Full Sample aPR (95% CI)	First-Trimester aPR (95% CI)	*p* for Interaction
H1	Lower educational attainment	1.24 (1.15–1.35)	1.20 (1.08–1.34)	0.312
H1	Lower household income	1.18 (1.09–1.27)	1.14 (1.03–1.27)	0.428
H1	Employment status	1.03 (0.96–1.11)	1.02 (0.90–1.15)	0.611
H2	Long mobile phone use	1.30 (1.20–1.41)	1.25 (1.10–1.42)	0.184
H2	Long TV sitting	1.25 (1.16–1.35)	1.20 (1.06–1.36)	0.247
H2	Avoided planned light activity	1.22 (1.14–1.31)	1.18 (1.05–1.32)	0.326
H2	Felt too tired/unmotivated	1.21 (1.13–1.30)	1.16 (1.03–1.31)	0.401
H3	<5 fruit/vegetable portions	1.36 (1.24–1.49)	1.30 (1.12–1.50)	0.218
H3	Sugar-sweetened beverages	1.27 (1.16–1.38)	1.21 (1.06–1.39)	0.337
H3	Fast-food intake	1.24 (1.14–1.35)	1.19 (1.04–1.36)	0.291
H3	Skipping breakfast	1.14 (1.05–1.24)	1.10 (0.98–1.24)	0.463
H3	Large portion sizes	1.05 (0.96–1.15)	1.03 (0.91–1.16)	0.702

## Data Availability

The original contributions presented in this study are included in the article. Further inquiries can be directed to the corresponding author.

## Abbreviations

Following abbreviations are used in this manuscript:

BMI	Body Mass Index
WC	Waist Circumference
VFR	Visceral Fat Rating
SES	Socioeconomic Status
SEM	Social Ecological Model
MoH	Ministry Of Health
GWG	Gestational Weight Gain
CI	Confidence Interval
aPRs	Adjusted Prevalence Ratio
UEBs	Unhealthy Eating Behaviours
